# Mapping the Qualitative Evidence Base on the Use of Research Evidence in Health Policy-Making: A Systematic Review

**DOI:** 10.34172/ijhpm.2020.201

**Published:** 2020-11-01

**Authors:** Ben Verboom, Aron Baumann

**Affiliations:** ^1^Centre for Evidence-Based Intervention, University of Oxford, Oxford, UK.; ^2^Swiss Tropical and Public Health Institute, Basel, Switzerland.; ^3^University of Basel, Basel, Switzerland.

**Keywords:** Evidence-Informed Policy-Making, Evidence Use, Qualitative Research, Systematic Review, Evidence-Informed Policy, Research Utilization

## Abstract

**Background:** The use of research evidence in health policy-making is a popular line of inquiry for scholars of public health and policy studies, with qualitative methods constituting the dominant strategy in this area. Research on this subject has been criticized for, among other things, disproportionately focusing on high-income countries; overemphasizing ‘barriers and facilitators’ related to evidence use to the neglect of other, less descriptive concerns; relying on descriptive, rather than in-depth explanatory designs; and failing to draw on insights from political/policy studies theories and concepts. We aimed to comprehensively map the global, peer-reviewed qualitative literature on the use of research evidence in health policy-making and to provide a descriptive overview of the geographic, temporal, methodological, and theoretical characteristics of this body of literature.

**Methods:** We conducted a systematic review following PRISMA (Preferred Reporting Items for Systematic Reviews and Meta-Analyses) guidelines. We searched nine electronic databases, hand-searched 11 health- and policy-related journals, and systematically scanned the reference lists of included studies and previous reviews. No language, date or geographic limitations were imposed.

**Results:** The review identified 319 qualitative studies on a diverse array of topics related to the use of evidence in health policy-making, spanning 72 countries and published over a nearly 40 year period. A majority of these studies were conducted in high-income countries, but a growing proportion of the research output in this area is now coming from low- and middle-income countries, especially from sub-Saharan Africa. While over half of all studies did not use an identifiable theory or framework, and only one fifth of studies used a theory or conceptual framework drawn from policy studies or political science, we found some evidence that theory-driven and explanatory (eg, comparative case study) designs are becoming more common in this literature. Investigations of the barriers and facilitators related to evidence use constitute a large proportion but by no means a majority of the work in this area.

**Conclusion:** This review provides a bird’s eye mapping of the peer reviewed qualitative research on evidence-to-policy processes, and has identified key features of – and gaps within – this body of literature that will hopefully inform, and improve, research in this area moving forward.

## Background

 The relationship between the worlds of scientific research and public policy has long been a preoccupation of social scientists.^1-3^ During the past few decades the widespread popularity of evidence-based medicine, which calls for the explicit, judicious and conscientious use of up-to-date research evidence in clinical decision-making,^4^ has accelerated discussion, debate and research on the role of research evidence in informing health policy decision-making.

 Proponents of evidence-informed policy-making in health assert that studies of various kinds can be used to address a range of questions of relevance to health policy-making.^5,6^ Perhaps most obviously, impact evaluations – including randomized controlled trials, quasi-experiments and other evaluation designs – can provide information on whether and to what extent a given policy or program is likely to be effective, and can therefore aid in the identification of, and adjudication between, competing policy alternatives. Moreover, evidence from both qualitative and quantitative research can help policy-makers to set policy agendas, by identifying, defining and prioritizing policy problems, and understanding and taking into consideration the perceptions of citizens, patients and other stakeholders. Finally, evidence can be drawn upon to identify and systematically account for potential factors affecting the implementation and scaling of policy interventions. Therefore, so it is often argued, research evidence can serve useful functions in various decision-making “stages” within policy processes – most commonly summarized as agenda setting, policy formulation, policy implementation, and policy evaluation^7,8^ – the key assumption being that health policy decisions which are informed by evidence are better than they otherwise would be in the absence of evidence.^9^

 However, as was argued in Carol Weiss’s pioneering work 4 decades ago,^10^ many – if not most – actual instances of “research utilization” in public sector bodies do not take the form of the direct, *instrumental* translation of research findings into discrete policy decisions, as models of evidence-based decision-making prescribe. Rather, the influence of research on policy is more often *conceptual, *following a gradual process through which the ideas that emerge from (social) science indirectly shape ways of thinking in policy circles, a process that Weiss termed enlightenment.^11^ Nor is research use necessarily a positive or desirable outcome: evidence can be drawn upon selectively to serve *symbolic *functions, for instance the legitimation of pre-existing political agendas^12^ or the justification of political inaction on the grounds that the existing evidence is insufficient.^13^ In such cases the “consideration” of the evidence by decision-makers might well follow the decision in question, not the other way around. Uses of research evidence can therefore be understood to serve not just instrumental (eg, problem-solving) functions, but also conceptual (eg, enlightenment) and symbolic (eg, political) functions in policy-making processes.^14^

 The point here is that the relationship between research evidence and public policy-making – far from the idealized straightforward, linear connection implied in some models – is highly contingent and complex, and can take various forms. It is perhaps no surprise, then, that interpretive qualitative methods are commonly suggested as particularly important to building our understanding of evidence-to-policy processes. Indeed, as argued by Contandriopoulos et al, the phenomenon of knowledge exchange is “ontologically more suited to case studies than to any other method” (p. 453), owing to the complexity of knowledge exchange interventions and to what they call the ‘systemic’ nature of the relevant outcomes, which frustrate attempts at valid quantitative measurement in this field.^15^

 Several systematic reviews focusing on various questions related to the use of research evidence by health policy-makers were published prior to the conduct of the present review.^16-21^ Three of these reviews summarized the literature on the barriers to and facilitators of evidence use, 2 in health policy specifically^19,20^ and one, most recently, in public policy more generally.^16^ A fourth review, originally published in 2011^17^ and subsequently updated in 2018^21^ extended beyond barriers and facilitators to examine a range of facets of decision-making in public health, but limited their included studies to those conducted in countries with universal healthcare systems (effectively excluding studies conducted in low-income countries, intergovernmental policy bodies, and the United States). Finally, Liverani and colleagues’ systematic review examined political and institutional influences on evidence use in public health policy.^18^ However, we know of no existing reviews that set out to provide a detailed mapping of these studies in order to paint a broad picture of their characteristics, nor any that were conducted with a specific focus on understanding the qualitative evidence base on the subject of evidence use by health policy-makers.

 The findings from these reviews suggest persistent academic interest in the subject of policy-maker evidence use, and indicate that the speed with which new primary studies on this topic are generated is rapidly growing. For example, roughly half of the 145 studies included in Oliver and colleagues’ review (spanning 2000-2012) were published in 2011 and 2012 alone.^16^

 While large and growing, the collection of research on the use of evidence by policy-makers – including the subset of this work that uses qualitative approaches – has long been subject to some common concerns and criticisms. From a methodological standpoint, it has been observed that this literature is dominated by the use of interviews and surveys to understand policy-maker perceptions about their use of evidence, with more direct methods of analyzing policy decisions, such as participant observation, sparsely deployed.^16^ Researchers have called for more in-depth, qualitative case studies of evidence use processes with attention to the important features of particular policy contexts, and for investigators to make greater use of more direct methods of observing policy-making activities using, for example, techniques commonly associated with ethnography.^22,23^ Another common refrain is that this literature is overwhelmingly preoccupied with addressing descriptive questions related to evidence uptake, most notably a disproportionate interest in the identification of barriers to and facilitators of the (instrumental) use of evidence, to the exclusion of more critical and explanatory concerns.^22,23^ Both within and beyond health-related research domains, barriers and facilitators conceptualizations have been criticized for oversimplifying complex social problems and for generating potentially misleading findings about how they might be overcome.^24-26^ Furthermore, the evidence-to-policy literature has been criticized for its theoretical naïveté,^27^ and in particular for its failure to harness theoretical and conceptual insights from political science and policy studies.^18,27,28^ In their 2013 review, Liverani and colleagues determined that only 6 of their 56 included studies “explicitly engaged with political theories or concepts.”^18^ Such neglect of political science has been identified as a weakness of academic public health more generally.^29,30^ In addition to these methodological and conceptual observations, concern has been raised that the research on evidence use is dominated by investigations from industrialized Western countries, and that as a consequence processes of evidence use in Global South are comparatively poorly understood.^31-35^ This paper, which reports the findings of an up-to-date systematic review of the qualitative academic literature on the use of research evidence in health policy-making, provides an empirical basis for some of these claims and concerns.

## Objectives

 The objectives of this review were: (1) to systematically map the global, peer-reviewed qualitative literature on the use of research evidence in health policy-making; and (2) to provide a descriptive overview of the studies that make up this literature, with an emphasis on their temporal and geographic distribution, methodological features, and subject matter focus.

## Methods

 We conducted a systematic review of published qualitative research on the role of evidence in health policy-making. In this paper, we provide a descriptive overview of this body of literature. The original protocol for the broader project of which this review is a part was registered with the International Prospective Register of Systematic Reviews (PROSPERO; Record CRD42018087940) and published elsewhere.^36^ The present review has been reported according to the PRISMA (Preferred Reporting Items for Systematic Reviews and Meta-Analyses) guidelines.^37^

###  Criteria for Considering Studies for This Review

 In this section we outline the criteria against which studies were assessed for inclusion in the review. Briefly, to be included a study had to:

- be a qualitative study published in a peer-reviewed journal;
- examine the work of *policy-makers* in *policy-making* settings; and
- report data concerning the use of *research evidence* to inform *health policy-making.*

In the sub-sections that follow, we provide a more detailed explanation of and rationale for these inclusion criteria.

####  Types of Studies

 This review includes primary qualitative studies published in peer-reviewed academic journals. We used the following definition of ‘qualitative study’: a study that uses qualitative methods both for data collection and data analysis. This definition is consistent with that used in several recent qualitative syntheses^38-40^ and was cited as one useful definition in the Cochrane Qualitative and Implementation Methods Group supplementary guidance on qualitative evidence synthesis.^41^ Methods of qualitative data collection include (but are not limited to) interviews, focus groups, and (participant) observation methods. Methods of qualitative data analysis include, for example, thematic analysis, phenomenological approaches, and grounded theory. This definition excludes studies in which data are collected through interviews or focus groups, but are analyzed exclusively through quantitative methods. To meet these methodological criteria, study authors had to explicitly describe the sources of data on which they drew. We considered studies to have used a qualitative method of data analysis if they used an identifiable term (eg, framework analysis) or citation to refer to the approach, or if it was clear that their procedures corresponded to a recognized method of qualitative analysis.

 We included mixed methods studies, that is, studies using both qualitative and quantitative methods, provided it was possible to examine the data derived only from the qualitative methods separately from the quantitative data, and where the qualitative component of the study corresponded to our subject matter inclusion criteria. We did not exclude studies according to the epistemological assumptions and/or theoretical traditions on which they were based. That is, we included all work within the broad qualitative paradigm.

 We did not exclude studies on the basis of a hierarchy of qualitative evidence or any other criteria related to study quality. It is not uncommon in reviews of quantitative research to impose a methodological quality “cut-off” based on features related to internal validity. However, the place of quality appraisal in qualitative reviews remains contentious,^41^ and no such cut-off criteria have found consensus among qualitative reviewers.^42,43^ Moreover, since the intention of the present review was to *exhaustively* catalogue and describe the published qualitative literature in this area (irrespective of any notion of quality), excluding relevant papers on the basis of quality would have been counter to our review objectives.

####  Types of Participants and Settings 

 This review includes studies involving policy-makers engaged in policy-making activities with an explicit (though not necessarily exclusive) focus on health issues. For the purposes of this review, the population ‘policy-makers’ includes elected officials, appointed civil servants, policy advisors and/or bureaucrats of any rank, working at the local, provincial/state, national, or supranational (ie, global/international) levels. Like other researchers in this topic area^44^ we found that reporting limitations in many interview studies on evidence use often made it difficult to identify the specific professional roles and activities of informants. We therefore excluded studies where it was impossible to determine with confidence that the actors or activities under study were policy-related.

####  Subject Matter of Studies

 In order to be eligible for inclusion, studies had to explore the use of *research evidence* by policy-makers working at least in part on *health policy*.

 For the purposes of this review, health policy decisions are those taken with the explicit goal of promoting population health and/or having to do with the financing and organization of health systems. We took policy-making to refer mainly to governmental planning and strategic decision-making about the organization of health services and public/population health, in contrast to public health management and practice. This excludes decisions related to patient-level, clinical healthcare or clinical governance. This implied distinction between policy actors, on the one hand, and those involved in management (eg, program managers, healthcare executives, and management consultants, with supervisory and management responsibilities in healthcare and public health organizations) and service delivery (eg, front-line practitioners, including nurses and physicians), on the other, is in line with previous reviews.^45^ Recognizing that policy decisions made outside of governmental health authorities, across a variety of policy sectors, can have meaningful impacts on health,^46^ we included studies in non-health sectors, as long as population health – or the relationship between policy decisions and health outcomes – was a major and explicit focus of the research or of the policy(ies) it examined.

 We defined *research evidence* as research produced by academic researchers and/or published in academic journals. This definition is similar to that used in a previous systematic review,^47^ whose authors found that their original attempt to use a broader definition of research evidence produced results so conceptually heterogeneous that a meaningful synthesis was unfeasible. This definition excludes studies that look exclusively at the use of raw data (eg, routine monitoring and surveillance data) by decision-makers. Eligible studies could have examined the use of research evidence in general, a specific methodological category of research (eg, randomized controlled trials, systematic reviews or other study types) or a particular form of research evidence (eg, evidence ‘embedded’ within written or verbal policy advice, including briefs, advisory reports, presentations and guidelines). The focus on evidence use had to be significant (ie, a core focus of the study) and explicit (eg, stated in the study’s research questions or objectives).

###  Search Methods for Identification of Studies

 We electronically searched a broad array of bibliographic databases (listed inBox 1**)** on January 20, 2019 using search strategies that were developed in consultation with information retrieval specialists and were subjected to multiple stages of piloting. We improved the sensitivity of each subsequent iteration of our search strategies by assessing detection of a list of key papers that were included in previous reviews on evidence use in policy-making. Strategies were iteratively amended (mainly through the addition of search terms and novel combinations of search terms) until all of these key papers were captured. Where appropriate, we adapted and applied methodological search filters to aid in the identification of qualitative studies.^48^ Our Medline search strategy is provided in Supplementary file 1.

**Box 1. Study Sources for Systematic Review**

** Bibliographic databases (no date/language limitation):**

- Applied Social Sciences Index and Abstracts
- Conference Proceedings Citation Index – Social Science and Humanities
- Global Health
- International Bibliography of the Social Sciences
- International Political Science Abstracts
- MEDLINE
- SCOPUS
- Social Sciences Citation Index
- Worldwide Political Science Abstracts

** Journals (January 2010 to January 2019):**

- BMC Health Services Research
- BMC Public Health
- Evidence and Policy
- Health Policy
- Health Policy and Planning
- Health Research Policy and Systems
- Implementation Science
- International Journal of Health Policy and Management
- Journal of Health Politics, Policy and Law
- Milbank Quarterly
- Social Science and Medicine

To offset the inevitable imperfections of electronic database searches, we also sought published studies through other search methods, including journal hand-searching, scanning reference lists, and speaking to experts. We hand-searched all issues of 11 relevant academic journals published from January 2010 to January 2019 (inclusive). Journals were selected for hand-searching on the basis of (1) their central relevance to the topic of the review (eg, *Evidence and Policy*), (2) our knowledge of their record of having previously published several relevant studies in this topic area (eg, *BMC Public Health*), and (3) advice from expert reviewers of early versions of this review’s protocol (eg, *Social Science and Medicine*). We also searched the reference lists of all included studies and of previous reviews whose subject matter focus had similarities with the present review.^16-21,47,49^ Experts and colleagues were contacted to obtain information about any as yet unidentified studies. Furthermore, we screened an inventory of studies of evidence-to-policy processes of which we were already aware.

###  Data Collection and Analysis 

 In this section we describe the methods for selecting studies, extracting and managing data, and analyzing and presenting the review findings. Both authors (BV and AB) conducted many of these tasks in parallel. Such double-screening, and double-extraction is standard practice in systematic reviewing,^50,51^ and is designed to limit the potential influence of bias and human error. In this review we treated the individual research report as the unit of analysis. We therefore use the terms ‘study,’ ‘article’ and ‘paper’ interchangeably. We used EndNote X9 software to manage references.

####  Selection of Studies

 Study screening and selection were conducted according to standard systematic review methods^50^ using Covidence systematic review software. BV and AB independently screened all titles and abstracts. Records deemed potentially relevant by both authors were retained for further review. Conflicting judgements were resolved through discussion. Since our aim was to comprehensively locate all studies meeting our inclusion criteria, and because it was often impossible to assess all inclusion criteria with confidence on the basis of titles and abstracts alone, we were deliberately very inclusive at this stage of screening. This was necessary, in large part, because of poor reporting of methodological information in qualitative study abstracts, as well as the ubiquity of relevant terminology (eg, “evidence-based policy”) in the titles and abstracts of papers with little relevance to the study of evidence use. As a result we retained a large number of papers for full-text review (see below).

 Both authors then independently screened the full text versions of all potentially relevant articles for inclusion in the review. All studies deemed to have met the inclusion criteria were included. Again, disagreements were resolved through discussion. Deferral to a third party to resolve disagreements on inclusion decisions was not necessary at either stage. Reasons for the exclusion of studies at the full-text review stage were recorded.

####  Data Extraction and Management

 A bespoke data extraction sheet was designed in Microsoft Excel to meet the specific objectives of the review. The following descriptive information was recorded for all included studies:

- Basic study information (authors, title, journal, year of publication);
- A brief summary of the study’s aim and research questions, and whether the concepts of ‘barriers to’ and/or ‘facilitators of’ the use of evidence were used in the study;
- Study design, description of data sources and qualitative analysis methods, theories or frameworks used for data collection and/or analysis;
- Description of the study setting, policy-making context, level of policy-making (ie, sub-national, national and/or international/global), and country or countries of focus;
- Description and number of participants;
- Description of the policy decision(s) or process(es) and policy sector(s) investigated;
- Type or form of research evidence investigated, and whether the study investigated instrumental, symbolic, and/or conceptual uses of evidence.

We first independently piloted the data extraction sheet on 30 included studies, which were selected at random. AB and BV compared the extracted data and resolved differences by discussion. During a second phase of piloting we extracted an additional 20 studies in duplicate to further enhance consistency. The remaining studies were divided between BV and AB for independent data extraction. Extractions conducted by AB were double-checked by BV to ensure consistency.

####  Data Analysis

 Data were tabulated and described narratively. Where appropriate, counts, sums, percentages and means were calculated. Previous reviews, including from health policy and systems research,^52^ inspired some of the analyses and the presentation of findings. We used the World Bank’s classification system to divide countries into 4 income groups according to Gross National Income per capita.^53^ We used colour-coded maps generated using web-based freeware MapChart.net^54^ to visually represent both the absolute number of studies per country and the density of studies as a proportion of country population. For the latter calculation we drew on data from the United Nations Population Division.^55^

 In order to characterize the subject matter of the body of included papers, each study was coded with a single ‘core’ primary focus, thrust or purpose, through an iterative, inductive process, following methods described in Erasmus and colleagues’ review of policy implementation research.^52^ Additionally, we coded all studies according to whether or not they sought to identify ‘barriers to’ and/or ‘facilitators of’ evidence uptake, regardless of whether this was the study’s core purpose.

 We drew on a number of common frameworks in order to classify studies. To categorize studies according to the policy activities on which they focus, we used the stages heuristic,^56-58^ a well-known (if simplistic) conceptual device (also known as the ‘policy cycle’^59^) which divides the policy process into 4 discrete stages: agenda-setting, policy formulation, policy implementation, and policy evaluation. A popular typology of research use (described above) was used to code studies according to whether they investigated instrumental, symbolic and/or conceptual uses of evidence.^14^

## Results

**Figure 1:**
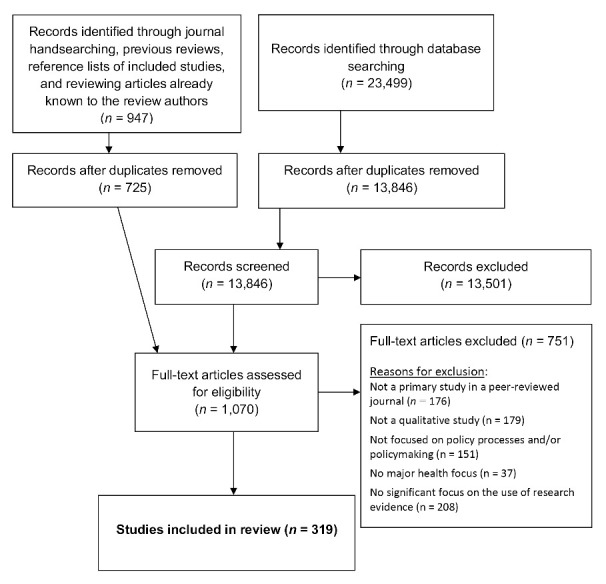
Diagram Showing the Flow of Studies Through the Review.

 The process of identification, screening, selection of studies in this review is summarized in the flow diagram in Figure 1. Nine-hundred forty-seven papers were identified by means of: consulting the included studies of previously-conducted reviews, journal hand-searching, scanning of reference lists of included studies, and by reviewing a list of potentially relevant studies of which we were already aware. Of these 947 articles, 725 were unique, and their full-text versions were retrieved and retained.

 Database searches yielded 23 499 records, 13 846 of which remained after manual and software-supported removal of duplicate records. Title and abstract screening of these records identified 345 additional potentially relevant and unique articles, bringing the total number of unique papers for full-text review to 1,070. Following full-text review, a total of 319 papers were found to meet our inclusion criteria (see Supplementary file 2 for the full list of included studies).

 The basic characteristics of the included studies are outlined in Table 1. All but 2 articles – one in Portuguese^60^ and one in Spanish^61^ – were published in the English language.

**Table 1:** Characteristics of Included Studies

**Domain**	**Category**	***N*** ^a^	**%** ^b^
Year of publication	Before 2000	11	3
	2000-2009	67	21
	After 2009	241	76
Journal	*Evidence and Policy*	39	12
	*Health Research Policy and Systems*	36	11
	*Social Science and Medicine*	18	6
	*Health Policy and Planning*	13	4
	*BMC Public Health*	11	3
	*Health Policy*	10	3
	*Implementation Science*	8	3
	*BMC Health Services Research*	6	2
	*International Journal of Drug Policy*	5	2
	*Journal of Public Health*	5	2
	*PLoS One*	5	2
	Other	163	51
Thematic focus of journal	Health	142	45
	Health and policy	100	31
	Policy	55	17
	Neither health nor policy	22	7
Study location by continent	Europe	118	37
	Africa	101	32
	Asia	70	22
	North America	69	22
	Oceania	46	14
	South America	12	4
Study location by country income classification	High-income	235	74
	Upper-middle-income	49	15
	Lower-middle-income	79	25
	Low-income	53	17
Countries most frequently investigated	United Kingdom	62	19
	United States	40	13
	Australia	38	12
	Canada	25	8
	Uganda	20	6
	The Netherlands	14	4
	India	13	4
	Malawi	13	4

^a^ This table represents data from a total of *N *= 319 studies. Some characteristics can have more than one value per study (eg, studies that investigate more than one country). Therefore, the sum of absolute values (ie, *N*) per domain can exceed the number of included studies and may vary between the domains. ^b^ Because percentages are rounded for each category of a domain, the sum of percentages per domain can slightly deviate from 100% (for characteristics that have one value per study).

###  Time Trends in Study Publication

**Figure 2:**
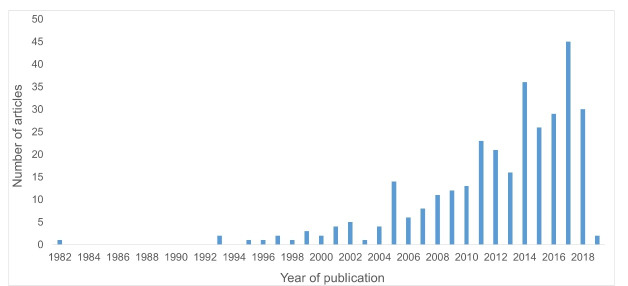
Number of Studies by Year of Publication.

 Our results indicate that publication of qualitative studies examining research evidence use in health policy has increased exponentially during the past several years. Included papers were published between the years 1982 and 2019. The 5 calendar years that produced the greatest number of included studies were 2014 through 2018, that is, the 5 most recent full calendar years captured by our review. This trend is illustrated in Figure 2, in which we present the number of included papers by year of publication. As the graph shows, more than 3 quarters of the articles we identified (76%) were published during the approximately 10-year period prior to our searches.

###  Publishing Venue

 Most studies were published in journals that either focus on health (n = 142, 45%) or both health and policy (n = 100, 31%). A smaller number of studies was published in journals related to policy only (n = 55, 17%) or in journals that are not focused specifically on health or policy (n = 22, 7%). Forty-five percent (n = 141) of all studies were published in only 8 different journals, with *Evidence *&* Policy* and *Health Research Policy and Systems* together accounting for nearly a quarter (n = 75, 24%) of all included studies (see Table 1).

###  Regional and Country Settings 

**Figure 3:**
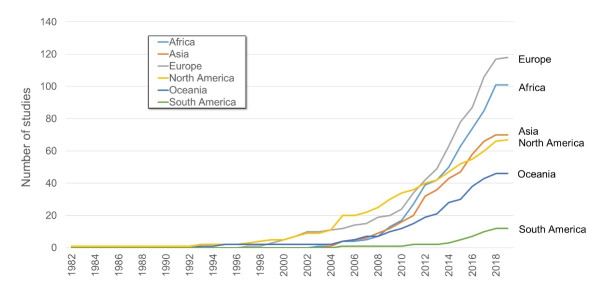
Number of Studies Per Continent Over Time (Cumulative).

 Included studies investigated policy processes and decisions in countries from every populated continent, with Europe (n = 118, 37%) and Africa (n = 101, 32%) being the most well-represented and South America (n = 12, 4%) relatively poorly represented. Europe and Africa are not only the most researched continents in absolute terms, but have also seen the greatest increase in research attention in this topic area during recent years (see Figure 3).

 A small number of included studies focused on country groupings or regions, or settings defined by other characteristics, as opposed to individual countries. These studies investigated decision-making within: the European Union (n = 3), the Eastern Mediterranean Region (n = 2), Caribbean Island States (n = 1), high-income countries (n = 1), low- and middle-income countries (n = 1), industrialized countries (n = 1), and within global multilateral organizations (n = 2).

 The 319 included studies investigated 72 distinct countries. Whereas 265 (83%) studies focused on a single country, 50 (16%) investigated more than one country, and 4 (1%) did not focus on a specific country or countries. Eight countries alone were studied in more than half (52%) of all included studies: the United Kingdom (n = 62, 19%), the United States (n = 40, 13%), Australia (n = 38, 12%), Canada (n = 25, 8%), Uganda (n = 20, 6%), the Netherlands (n = 14, 4%), India (n = 13, 4%), and Malawi (n = 13, 4%) (Table 1). The majority of studies were conducted, at least in part, in countries with high-income status (n = 235, 74%), while 15% (n = 49) were conducted in upper-middle-income countries, 25% (n = 79) in lower-middle-income countries, and 17% (n = 53) in low-income countries.

**Figure 4:**
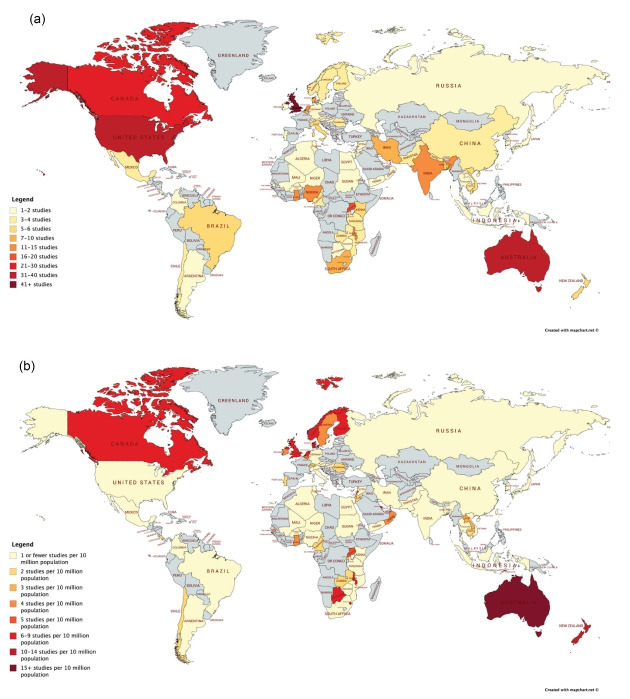
Maps of Countries Investigated in Absolute Numbers (a) and Per Unit Population (b).

 A visual depiction of the global distribution of included studies by country of focus is shown in Figure 4. The figure displays the absolute number of studies per country (4a) and the study density per country adjusted by population (4b). When adjusted for population size, the countries with the highest study density are (in decreasing order): Fiji, Australia, New Zealand, Denmark, the United Kingdom, Eswatini, Botswana, and the Netherlands.

###  Subject Matter of Studies

 All studies were assigned to a single category that best summarized their core purpose, focus or general thrust.^52^ These are summarized thematically in Table 2,alongside the number of studies falling into each category, as well as the number of studies in each category that drew on the barriers and facilitators concepts to address their research questions.

**Table 2:** Primary Purposes, Thrusts or Objectives, and Number of Studies Using Bs/Fs Concepts

**Category**	**Purpose, Thrust or Objective**	***N *** **(Total)**	***N *** **(Bs/Fs)**
**Cases of policy change or decision-making (88 studies)**			
Evidence-in-policy cases	To examine the role of evidence in a specific case or cases of policy change or decision-making	68	17
Broad policy-making cases	Studies broadly investigating a case of policy change or decision-making, with a partial but significant focus on the influence of research	20	3
**Studies focused on the use or impact of specific pieces or categories of evidence (53 studies)**			
Methodological category of evidence	To examine the usefulness or use of a particular methodological category of evidence (eg, economic evaluations)	20	13
Specific piece(s) of evidence	To assess the impact or use of specific piece(s) of evidence (eg, specific studies) in policy decisions	13	5
Topical categories of evidence	Studies on the use or usefulness of evidence in a specific topical or thematic area (eg, social determinants of health)	7	2
Specific body of evidence	To assess the impact or use of a specific body of evidence (eg, outputs from a research program) in policy decisions	6	1
Embedded evidence	Studies assessing the use of evidence embedded in a specific format or “vehicle” (eg, policy guidance)	5	1
Foreign evidence	Studies on the use of evidence from other countries	2	0
**Perceptions and preferences studies (36 studies)**			
General perceptions	General perceptions of policy-makers (as well as researchers and other stakeholders) on the use of evidence in policy	20	11
Evidence-related needs	To identify the research evidence needed by a particular group of policy-makers, and/or their needs in relation to how evidence should be communicated or delivered	7	2
Preferred types of evidence	Policy-maker perception of types of research (eg, methodological or thematic categories) that are useful	6	0
Sources of evidence	To understand the main sources (eg, databases, contacts) through which policy-makers access evidence	3	0
**Institutional, political and organizational contexts (36 studies)**			
Political and governance contexts	To investigate how political contexts and circumstances, and/or governance arrangements influence evidence use in policy	11	1
Institutional arrangements for evidence use	Studies that investigate the role of institutional structures specifically designed to support evidence use in policy (eg, knowledge transfer units within government)	9	0
Climate for evidence use	Studies seeking to understand the “climate for evidence use” in a policy context (eg, a country or other jurisdiction)	7	4
Everyday decision-making	To understand how decisions are made in day-to-day practice in a policy organization, including the role of research evidence	6	0
Organizational capacities for evidence use	To understand the organizational-level capacities, capabilities and tools that facilitate the use of research evidence	3	2
**Methodological and theoretical contributions (27 studies)**			
Critical social science perspectives	Studies that problematize notions of evidence, or seek to critically reconceptualize the evidence-policy relationship	12	0
Evidence use models	To assess evidence use models against real-world policy-making	8	1
Complexity and systems approaches	Studies that apply and/or explore the explanatory value of methods derived from complexity theory or systems thinking to understand the dynamics of evidence-to-policy processes	4	0
Novel methods	To demonstrate a novel qualitative method for studying evidence use in policy	3	1
**Researchers, research organizations and other external stakeholders (24 studies)**			
Relationships, interaction and collaboration	Studies examining the influence of researcher-policy-maker interaction and collaboration on the use of evidence	9	4
Research organizations	Studies on the role of research organizations or bodies (eg, think tanks) in supporting evidence use	7	0
Researchers and their roles	To investigate the roles of researchers in, and their influence on, the policy process	3	0
External actors	To understand the role of external stakeholders (eg, NGOs) in facilitating evidence use in policy processes	3	0
Community-based participatory research	Studies exploring the influence of community-based participatory research partnerships on policy-making	2	0
**Factors (barriers and facilitators) studies (23 studies)**			
Factors affecting evidence use	To identify and catalogue factors related to evidence use (usually conceptualized as barriers and/or facilitators) in particular policy context(s)	23	23
**Intervention studies (9 studies)**			
Interventions to improve evidence use	Studies assessing the implementation, effects or participant experiences of an intervention for improving evidence use	9	5
**Other categories of studies (23 studies)**			
Communication and dissemination	To examine dissemination of research to policy-makers and to improve research communication strategies and initiatives	6	2
Evidence-policy concordance	Studies aiming to assess and/or explain the (mis)alignment between documented policy positions, decisions or preferences, and the available evidence	6	1
Argumentation, debate and rhetoric	Studies examining political argumentation and/or the rhetorical uses of evidence in policy debates	5	0
Miscellaneous topics related to evidence in policy	Studies on a range of specific topics, including the role of research commissioning and commissioners, interplay between research evidence and traditional Aboriginal knowledge, influence of the media, the scaling up of evidence-based best practices, and the use of evidence in judicial decision-making	6	0

Abbreviations: Bs/Fs, Barriers and Facilitators; NGOs, non-governmental organization.

 The single largest category of studies were those whose core focus was to understand the role of research evidence in a specific case of policy change or decision-making (n = 68, 21%). A large number of studies (n = 53, 17%) were centred around a specific category of evidence – most commonly a methodological grouping (n = 20, 6%), or a specific study or studies (n = 13, 4%) – and sought to understand their impact on or use in policy decisions. Also common were studies focused on policy-maker perceptions – related to evidence use generally (n = 20, 6%), or more specifically to their evidence-related needs (n = 7, 3%) and preferred types (n = 6, 2%) and sources (n = 3, 1%) of evidence.

 Twenty-three studies (7%) were classified as having the identification of factors related to evidence use (ie, barriers and facilitators) as their core objective. However, overall, nearly one-third of studies (n = 99, 31%) investigated barriers and facilitators in some way, usually as one part of a broader set of study objectives.

###  Types of Study Designs and Methods Used

 The qualitative study designs and methodological features of included studies are provided in Table 3. Over half of all included studies can be described as case studies (n = 181, 57%), in that they set out to investigate a specific case – or set of cases – of policy decision-making, of research dissemination processes, of intervention roll-out or implementation, or other events. Other identifiable qualitative study designs (ie, ethnographies, evaluations and participatory action research) were used in only 6% (n = 18) of all studies. A large number of studies (n = 120, 38%) could not be identified according to particular qualitative study design and were therefore classified as “other” (these tended to include studies drawing solely on either interviews or documents, or cross-sectional surveys with qualitative components).

**Table 3:** Study-Level Characteristics Related to Study Design and Methods Used

**Domain**	**Category**	***N*** ^a^	**%** ^b^
Study design	Case study	181	57
	Ethnography	8	3
	Evaluation	8	3
	Participatory action research	2	1
	Other	120	38
Mixed methods	No	271	85
	Yes	48	15
Data sources	Interviews	282	88
	Documents	160	50
	Focus groups	34	11
	Observation	33	10
	Other	47	15
Data sources – single or multiple	Multiple	180	56
	Single	139	44
Qualitative analysis method	Thematic analysis	118	37
	Content analysis	49	15
	Grounded theory approaches	31	10
	Framework analysis	24	8
	Phenomenological approaches	8	3
	Discourse analysis	6	2
	Narrative analysis	1	<1
	Other	23	7
	Unclear/Not described in detail	59	18

^a^ This table represents data from a total of *N *= 319 studies. Some characteristics can have more than one value per study (eg, studies that used more than one data source). Therefore, the sum of absolute values per characteristic (ie, *N*) can exceed the number of included studies and may vary between the characteristics. ^b^ Because percentages are rounded for each category of a domain, the sum of percentages per domain can slightly deviate from 100%.

 Forty-eight articles (15%) reported mixed methods studies that used both qualitative and quantitative approaches, whereas the vast majority of studies (n = 271, 85%) relied exclusively on qualitative methods. The overwhelming majority of studies drew on interviews (n = 282, 88%) and/or documents (n = 160, 50%), while focus groups (n = 34, 11%) and methods of observation (n = 33, 10%), were less commonly used. Well over half of all studies (n = 180, 56%) combined multiple sources of qualitative data, with interviews and documents being by far the most common combination.

 Nearly two-fifths of included papers described using thematic analysis (n = 118, 37%), followed by content analysis (n = 49, 15%), grounded theory approaches (n = 31, 10%), framework analysis (n = 24, 8%), phenomenological approaches (n = 8, 3%), (critical) discourse analysis (n = 6, 2%), and narrative analysis (n = 1, <1%). We found that the methods of data analysis used in included studies were in many cases not well-described. Almost a fifth of all studies (n = 59, 18%) did not report their analysis at all or were unclear in their reporting of how it was performed, while a minority of studies (n = 23, 7%) described their analysis only in generic or broad terms. Even where reporting of analysis methods bordered on satisfactory, it was still often difficult to categorize. For instance, in the case of studies using what we determined to be a form of thematic analysis almost half (n = 58, 18%) described their procedures without explicitly referring to thematic analysis or a related label, necessitating a degree of judgement on our part.

###  Use of Theory and Frameworks

 Studies used various theories and frameworks to investigate evidence use in policy, as reported in Table 4. Almost half of all studies applied a theory or framework to inform data collection or analysis (n = 156; 49%). Twenty-two percent (n = 71) of studies used theories or conceptual frameworks drawn from or based on political science or policy studies. Over half (n = 163, 51%) of all papers did not report the use of any theory or conceptual framework.

**Table 4:** Use of Theories and Frameworks in Included Studies

**Category**	***N*** ^a^	%
Use of theories and frameworks		
Studies using a policy/political theory or framework	71	22
Studies using other type of theory or framework only	85	27
Studies using no theory/framework	163	51
Theories and frameworks by frequency of use		
Multiple Streams Theory (John Kingdon)	19	6
Typology of Research Utilization (Carol Weiss)	18	6
Policy Triangle (Gill Walt and Lucy Gilson)	16	5
3-Is Framework (Interests, Ideas and Institutions)	6	2
ODI RAPID Framework (Context, Evidence and Links)	5	2
Pathways to EIPP Framework (Bowen and Zwi)	5	2
Other theories/frameworks (used in <5 studies)	108	34

Abbreviations: RAPIR, Research and Policy in Development; EIPP, ‘evidence-informed’ policy and practice. ^a^ This table represents data from a total of *N* = 319 studies. Some studies used more than one theory or framework, thus the sum of specific theories/frameworks (ie, *N*) exceeds the number of included studies that used at least one theory/framework.

 The most commonly used theories and conceptual frameworks are reported in the bottom half of Table 4. Only 6 theories or frameworks were used in 5 or more papers. The 3 most popular theories/frameworks among our included studies were Kingdon’s Multiple Streams theory (n = 19, 6%), Weiss’s research utilization typology (n = 18, 6%), and Walt & Gilson’s ‘Policy Triangle’ (n = 16, 5%).

###  Types of Study Participants

 Included studies that involved participant responses (defined here as having used either individual interviews, focus groups or a combination thereof) and that reported the number of study participants (n = 264, 83%), investigated a total of 9436 participants. Of these, 8595 (mean = 34) were interview (as opposed to focus group) participants. However, many studies did not report details of the participants and their numbers sufficiently enough to be included in these calculations: in 30 (9%) studies the overall number of participants was not clearly reported; nearly half of studies that drew on participant responses did not provide sufficient information to determine the number of participants who were policy-makers (n = 142, 45%). Among those studies in which it was possible to make such a determination, 60% (2973) of participants were identified as some kind of policy-maker.

 Where possible, we attempted to distinguish between studies that included political decision-makers (eg, elected politicians) and non-political policy-makers (eg, civil servants, bureaucrats, policy advisors) among their participants. The majority of studies (n = 167, 52%) exclusively included non-political policy-makers, while one quarter (n = 82, 26%) focused on both groups. Only 5% (n = 16) of studies that included participants exclusively targeted politicians. In 54 (17%) of this review’s included studies, authors did not provide sufficient information to determine the types of policy-makers who were interviewed.

###  Policy and Governance Features

 We categorized included studies according to several policy- and governance-related characteristics. This analysis is summarized in Table 5.

**Table 5:** Features of Included Studies Related to Policy-Making and Policy Sector

**Domain**	**Category**	***N*** ^a^	**%**
Level of policy decision-making	Supranational	7	2
	National	188	59
	Sub-National (any)	139	44
	Provincial or state (or equivalent)	79	25
	Local, regional or municipal	75	24
	No specific focus	29	9
Policy stage	Agenda setting	41	13
	Policy formulation	145	45
	Policy implementation	35	11
	Policy evaluation	8	3
	Not focused on a specific stage (or stage unclear)	161	50
Policy sector	Public Health	189	59
	Healthcare	156	49
	Criminal justice and law enforcement	10	3
	Transportation	8	3
	Education	5	2
	Environment	5	2
	International development	5	2
	Agriculture, Food and Nutrition	4	1
	Social care	3	1
	Child welfare and protection	3	1
	Housing	2	1
	Urban planning	2	1
	Social services	1	<1
	Labour and employment	1	<1
	Several sectors or no specific sector	8	3

^a^ This table represents data from a total of N= 319 studies. Some characteristics can have more than one value per study (eg, studies that investigated more than one policy level). Therefore, the sum of absolute values per characteristic (ie, N) can exceed the number of included studies.

 Well over half of all included studies were concerned, at least in part, with policy-making at the national level (n = 188, 59%). One-hundred thirty-nine studies examined sub-national (41%) policies or policy-making, with 79 (25%) of these studying provincial or state (or equivalent) decision-making, and 75 (24%) studying local-level (ie, municipal or regional) policy-making. Seven studies (2%) were concerned with policy-making at the supranational level. These studies examined the use of evidence in decision-making within the European Union, World Health Organization (WHO), and other international policy fora. A considerable number of studies (n = 29, 9%) investigated the perspectives of policy-makers in general without focusing on a particular policy or level of governance.

 Of the studies that focused on a specific stage or stages of the policy process, we found that most (n = 145, 45%) examined policy formulation, either alone or in addition to other stages. A roughly similar number of studies focused on agenda-setting (n = 41, 13%) and policy implementation (n = 35, 11%), while few focused on policy evaluation (n = 8, 3%). Overall we found that the focus of most studies could not be summarized under the heading of a policy stage (n = 161, 50%). Many of these studies investigated a policy process holistically, or policy-making in general within a particular field, without distinguishing between various policy activities.

 Unsurprisingly, given the health focus of this review, a great majority of studies looked at policies or policy-making activities within the sectors of public health (n = 189, 59%), healthcare (n = 156, 49%) or both of these. However, a significant number of these studies investigated health-related policies or policy processes that also had relevance in non-health sectors, including criminal justice and law enforcement (n = 10, 3%), transportation (n = 8, 3%), education (n = 5, 2%), environment (n = 5, 2%), and international development (n = 5, 2%).

 Many studies did not describe in detail (if at all) what they understood by the terms “policy” or “policy process.” Many studies termed their focus “policy(making) and practice,” but provided no definitions for, or otherwise distinguished between, these 2 concepts. This was especially the case with studies that examined local levels of policy-making.

###  Evidence- and Research-Related Features

 All studies included in this review focused in some way on the use of academic research evidence. However, whereas some studies focused specifically on research evidence, others considered research alongside other forms of evidence. That is, some studies investigated research use in the context of broader investigations of knowledge or other kinds of evidence. Conversely, many other studies took a more specific focus, studying either a specific methodological category or other type of research evidence (eg, systematic reviews), while others still were specifically concerned with what we called evidence “formats,” that is to say, evidence embedded in or communicated via particular vehicles (eg, reports, guidelines). The research evidence focus of included studies, as described by their authors, is summarized in Table 6.

**Table 6:** Research Evidence Focus of Studies

**Domain**	**Category**	***N*** ^a^	**%**
Type of evidence investigated	Research evidence or category thereof	247	77
	Research evidence (in general)	177	55
	*Particular type of research*		
	Economic evaluations	12	4
	Systematic reviews	8	3
	Health technology assessments	8	3
	Evaluation studies	7	2
	Randomized controlled trials	7	2
	Models or modelling studies	6	2
	Surveys	3	1
	Burden of disease information	2	1
	Health impact assessments	2	1
	Operational research	1	<1
	Community-based participatory research	1	<1
	Population health rankings	1	<1
	Needs assessments	1	<1
	*Particular forms/formats of embedded research*		
	Reports	4	1
	Guidelines or recommendations	4	1
	Evidence services	2	1
	Evidence summaries	1	<1
	Broad focus on ‘knowledge’ in general	72	23
Functional evidence use categories	Instrumental uses of evidence	183	57
	Symbolic uses of evidence	64	20
	Conceptual uses of evidence	43	13
	No specific or discernible focus	122	38
Combinations of functional evidence use categories	Instrumental use only	122	38
	Instrumental + symbolic + conceptual uses	32	10
	Instrumental + symbolic uses	22	7
	Symbolic use only	10	3
	Instrumental + conceptual uses	7	2
	Conceptual use only	4	1
	Conceptual + symbolic uses	0	0

^a^This table represents data from a total of *N *= 319 studies. Studies can have more than one evidence type focus, thus the sum of health topics (ie, *N*) exceeds the number of included studies.

 Most studies (n = 247, 77%) had a clear, central focus on research evidence or a category (type or format) thereof, as opposed to those that studied ‘knowledge’ or ‘evidence’ more generally (n = 72, 23%), in which the use of other kinds of knowledge (eg, tacit knowledge) might be studied alongside the use of research evidence. However, it is worth noting that what the study authors subsumed under the terms “evidence,” “research” and “research evidence” differed greatly between the studies. One fifth of all studies (n = 70, 22%) focused on a particular type of research evidence. Of particular interest was the use of economic evaluation (n = 12, 4%), systematic reviews (n = 8, 3%), health technology assessment (n = 8, 3%), evaluation studies (n = 7, 2%), randomized controlled trials (n = 7, 2%), and modelling studies (n = 6, 2%). A small number of studies looked at the use of evidence packaged in different delivery formats, including reports (n = 4, 1%), guidelines or recommendations (n = 4, 1%), and information from evidence ‘services’ (n = 2, 1%) and summaries (n = 1, <1%).

 Regarding the functional categories of evidence use, instrumental use was investigated (alone or in combination) by 183 studies (57%), while symbolic and conceptual uses were investigated to a lesser extent, by 64 studies (20%) and 43 studies (13%), respectively.

 These categories appeared in a number of different combinations in included studies. While a large plurality of studies investigated instrumental uses only (n = 122, 38%), it was also not uncommon for instrumental and symbolic uses (n = 32, 10%), and conceptual, instrumental and symbolic uses (n = 22, 7%) to be studied in combination. Notably, very few studies investigated either symbolic (n = 10, 3%) or conceptual (n = 4, 1%) evidence use without also looking at instrumental uses.

## Discussion

 Qualitative research on the role of research evidence in health policy-making is a popular area of inquiry, and one that is rapidly expanding. In this systematic review, we sought to comprehensively assemble the qualitative evidence base that has investigated the use of research evidence in health policy-making. This review uncovered 319 published qualitative studies on evidence use in health policy spanning the period from 1982 to 2019. While a large proportion of these studies is still drawn from high-income regions like Western Europe, North America and Australia, a growing proportion of this topic area’s output is now coming from low- and middle-income countries, especially from sub-Saharan Africa.

 We found that a significant number of studies in this topic area – though by no means a majority – sought to catalogue ‘factors’ related to the use of research in policy, and that these studies conceptualized such factors as barriers to and facilitators of evidence uptake. We classified 23 studies as having this as their core objective and, overall, nearly 100 studies – almost one third of included studies – used the barriers and facilitators constructs in some way. While this finding does not contradict the received wisdom in this topic area that the identification of ‘factors affecting’^22^ and/or ‘barriers and facilitators’^23^ is one of the most well-travelled lines of inquiry, it does indicate that the cataloguing of such factors is usually not the sole or central focus of qualitative studies of evidence use.

 Indeed, as demonstrated in this review, the subject matter of these studies is quite varied. For example, we found that large numbers of studies drew on qualitative methods to study the role of research relative to other competing influences in cases of real-world policy change, to examine how evidence use is influenced by political and governance contexts, and to explore how researchers, research organizations and other external stakeholders influence processes of evidence use. Studies took on issues as diverse as, for instance, strategic uses of research evidence in service of political and corporatist interests,^62^ the phenomenon of “imposed” evidence use, in which decision-maker attention to research evidence is compelled through external pressure, top-down regulation, and the like,^63^ and even the performative “production” of the evidence-based policy paradigm itself,^64^ among a range of other topics.

 The literature on evidence-informed policy-making is sometimes said to lack in-depth, rich case studies on policy decisions and processes, and few attempts to study evidence use as it occurs in real-world practice through the use of observational methods.^22^ We found that many of the studies identified in this review drew on in-depth case studies – often comparative investigations across multiple countries – to examine how and why evidence was used, or not used, in specific instances of policy development or change. This may indicate a gradual shift in research priorities and approaches over time, including a trend toward more such in-depth policy case studies. Still, consistent with previous reviews,^16,18^ we identified very few studies that employed designs (eg, ethnography) and data collection methods (eg, participant observation) that involve direct, real-time observation of policy-making activities and decisions, and that do not primarily depend on eliciting retrospective perceptions in the context of a research interview or focus group.

 Many authors have lamented the theoretical shortcomings of the literature on evidence use in health policy, most notably the lack of engagement with political science and public administration theories and concepts.^18,27,28^ For instance, Liverani et al^18^ observed that studies in this area *“do not constitute a clearly defined body of research, developed around shared debates, research questions or theoretical approaches”* and that *“despite the fundamentally political nature of decision making processes [and] the extensive literature on political institutions…very few works could be identified which explicitly applied policy science perspectives to understand the use of evidence in health policy making”* (p. 6). Our review largely confirms this: we noted that while about half of studies used an identifiable theory or conceptual framework, there was a high degree of theoretical variability with no clear dominant approach. While it remains the case that most qualitative studies in this topic area do not explicitly contribute to the development of political science theories and the refinement of policy science concepts, we nevertheless identified a considerable number of studies that employ these theories and conceptual frameworks to guide their data collection and to make sense of their findings. This finding may indicate that calls for greater engagement with policy theories and political concepts are slowly beginning to be heeded by scholars of evidence-to-policy processes in health.

 A key finding of this review is that this literature focuses overwhelmingly on the use of research in the policy activities of technical – as opposed to political – decision-makers. The reasons for this are likely many, but it probably owes in part to the relatively high degree of availability of civil servants for research participation, as compared to political actors, as well as the fact that most engagement with the technical aspects of policy development – that are perhaps more amenable to instrumental and other direct forms of evidence use – is work done by unelected decision-makers working in government bureaucracies. Still, high-level policy decisions relevant to health systems and public health, including large budget allocations, decisions about system restructuring and healthcare reform, and even smaller scale decisions of a politically contentious nature, are taken with the direct participation of politicians. We also found that relatively few studies provided in-depth explorations of symbolic uses of evidence, that is, the marshalling of evidence, often selectively, to serve political or tactical ends (eg, to legitimate pre-existing political agendas). Moving forward, further research on how research evidence features in the decision-making of political actors, including such strategic uses of evidence, would help to provide a more complete picture of the relationship between research and policy processes.

###  Strengths and Limitations of This Review

 In this review, rigorous systematic review methods were used, including careful piloting of procedures in each review phase, strict double-screening and study selection, and quality assurance measures for data extraction. Multiple sources were searched for relevant studies and a highly sensitive bibliographic database search was developed and conducted across 9 databases. This review can therefore be considered a comprehensive collation of the published and peer-reviewed qualitative literature on evidence use in health policy.

 While inclusive and broad in many respects, this review also has a specific focus on health-related policy-making, which may entail some limitations. As suggested by Lorenc and colleagues’ review of evidence use in non-health sector policy decision-making,^47^ there may exist distinct and idiosyncratic evidential ‘cultures’ in different policy sectors. While the present review probably captures the majority of qualitative studies in the overall topic area of research evidence use in policy-making (given that the preponderance of evidence in this area comes from public health and healthcare policy) these findings are not necessarily generalizable to the evidence-to-policy topic area as a whole.

 Moreover, because of this project’s specific interest in qualitative evidence the review only considered qualitative (and qualitative-quantitative mixed methods) studies. Previous reviews demonstrate that a considerable amount of quantitative evidence exists in this topic area^16,19^ that may provide unique insights about evidence-to-policy processes that are inaccessible to qualitative research. The present review was not designed to capture these studies.

 We did not conduct any form of quality appraisal or ‘risk of bias’ assessment as part of this review. While we recognize that qualitatively synthesizing studies without consideration of methodological rigor has the potential to bias synthesis findings,^65^ the descriptive overview reported in this paper does not entail such a synthesis. We did not consider it worthwhile to subject all included studies to quality appraisal with a methodological checklist merely for the purposes of reporting study quality, especially since such instruments are not designed to generate a summary ‘score’ that serves as a standalone indicator of study quality, but instead are meant to function as a tool to facilitate a critical, engaged reading of a study’s methodological strengths and weaknesses.^41^

 Finally, for the purposes of this review, we chose to treat the individual research report (ie, article) – rather than the study, as is often the case in Cochrane-style reviews – as the unit of analysis. One consequence of this decision is that, in some cases, different reports from the same research project have contributed individually to the descriptive statistics. Thus, these statistics are influenced disproportionately by larger programs of research with comparatively high publication outputs. Given our interest in painting a general picture of the existing qualitative literature in this topic area (as opposed to, eg, conducting a meta-analysis) we did not consider this to be highly problematic.

## Conclusion

 This systematic review constitutes the most comprehensive mapping of the extant qualitative literature on the use of research evidence in health policy-making conducted to date. It has provided a “bird’s eye view” of this rapidly growing literature, and has identified key features of – and gaps within – this body of research that will hopefully inform future scholarship in this area.

 The use of research evidence in health policy processes is a burgeoning area of scholarship, and the qualitative literature on this subject is expanding with increasing speed year-on-year. Indeed, well over half of all of the qualitative studies on evidence-to-policy processes in health were published during the past 5 years alone. While high-income countries – especially Australia, Canada, the United Kingdom and the United States – still lead the qualitative research output in this area, the share of research coming from the Global South is growing. Over 100 qualitative studies on evidence use in African health policy have now been published, and the continent is second only to Europe in overall output.

 Qualitative researchers have investigated a diversity of sub-topics related to evidence use. This review has shown that, while certainly a major preoccupation of evidence-to-policy researchers in this area, barriers to and facilitators of evidence use are not the single dominant focus, at least among qualitative investigations. Attention may be shifting (if gradually) to less descriptive topics, with several examples of complexity science-informed approaches, explanatory case studies of policy processes, and critical social science investigations of the evidence-based policy paradigm, among many other topics, emerging from this review.

 While this literature is extensive, this review has identified some notable gaps that future qualitative literature should address. On the methodological front, there remain relatively few studies that draw on qualitative observational methods to investigate the interactions between research and policy in everyday policy activities. Our knowledge of how, why and under what circumstances policy-makers engage with, use, and/or misuse research would benefit from such immersive work by, for example, participant observers. As well, the vast majority of studies explore the role of civil servants and other unelected decision-makers in evidence-to-policy processes, with far less focus on politicians. Further qualitative study of the how political actors engage with evidence – especially, though not exclusively, how they deploy research-based claims for political, tactical and rhetorical purposes – would greatly enrich this literature.

## Ethical issues

 Not applicable.

## Competing interests

 Authors declare that they have no competing interests.

## Authors’ contributions

 BV conceived the study and designed and ran the electronic search strategies. AB supported the testing and refinement of the search strategies. Both authors developed the tools and procedures used, screened and selected studies for inclusion, extracted, managed and analyzed the data, and co-drafted the manuscript. Both authors read and approved the final manuscript.

## Funding

 There was no dedicated funding for this review. BV received financial support from the Pierre Elliott Trudeau Foundation. AB is financially supported by the Swiss State Secretariat for Education, Research and Innovation SERI (swissuniversities PgB-4) and the Swiss Tropical and Public Health Institute.

## Supplementary files

Supplementary file 1. Sample Search Strategy (MEDLINE).

Supplementary file 2. List of Included Studies.
