# Supplementary material for: Mapping the Qualitative Evidence Base on the Use of Research Evidence in Health Policy-Making: A Systematic Review
Source: Int J Health Policy Manag. 2020 Nov 1;11(7):883–98. doi: 10.34172/ijhpm.2020.201 (PMC9808178; doi:10.34172/ijhpm.2020.201)
Supplement: Supplementary file 1 — Sample Search Strategy (MEDLINE). [file ijhpm-11-883-s001.pdf]

Supplementary file 1. Sample Search Strategy

MEDLINE (Ovid)

(Run on 20 January 2019)

#	Searches	Results
1	Evidence Based Practice/ or ("research evidence" or "evidence based" or "evidence informed" or "knowledge translation" or "knowledge transfer" or "knowledge exchange" or "knowledge broker*" or "knowledge mobili?ation" or "using evidence" or "using research" or "using knowledge" or "using information" or "using science" or "using scientific" or ((evidence or research or knowledge) adj3 (use* or utilis* or utiliz* or uptake or diffus* or disseminat*)) or ((systematic review* or evaluation* or Technology Assessment* or HTA*) adj2 (use* or utiliz* or utilis* or uptake or diffus* or disseminat*))).tw.	208317
2	exp Public Policy/ or exp Policy Making/ or exp Government/ or Decision Making/ or Policy/ or Politics/ or Administrative Personnel/ or Government Employees/ or (health* policy* or health* policies or (health* adj2 planning) or (policy* adj2 decision*) or (political adj2 decision*) or (policy* adj2 develop*) or (policies adj2 develop*) or (policy* adj2 formulat*) or (policies adj2 formulat*) or policy mak* or policymak* or public policy* or public policies or policy* process* or policy* change* or legislat* or politician* or bureaucrat* or governance or (government* adj2 agenc*) or (government* adj2 policy*) or (government* adj2 policies) or (government* adj2 decision*)).tw.	499193
3	exp Health Policy/ or exp Health Planning/ or Health Services/ or Public Health/ or Health Promotion/ or (health* policy* or health* policies or health system* or healthcare system* or health care system* or health service* or (ministr* adj3 health*) or (department* adj3 health*) or (health* adj2 planning) or public health or population health or health promotion or health sector).tw.	828448
4	1 and 2 and 3	9619
5	Qualitative Research/ or Interview/ or (theme$ or thematic).mp. or qualitative.af. or Nursing Methodology Research/ or questionnaire$.mp. or ethnological research.mp. or ethnograph$.mp. or ethnonursing.af. or phenomenol$.af. or (grounded adj (theor$ or study$ or studies or research or analys?s)).af. or (life stor$ or women* stor$).mp. or ((emic or etic or hermeneutic$ or heuristic$ or semiotic$).af. or (data adj1 saturat$).tw. or participant observ$.tw.) or (social construct$ or (postmodern$ or post-structural$) or (post structural$ or poststructural$) or post modern$ or post-modern$ or feminis$ or interpret$).mp. or (action research or cooperative inquir$ or co operative inquir$ or co-operative inquir$).mp. or (humanistic or existential or experiential or paradigm$).mp. or (field adj (study or studies or research)).tw. or human science.tw. or biographical method.tw. or theoretical sampl$.af. or ((purpos$ adj4 sampl$) or (focus adj group$)).af. or (account or accounts or unstructured or open-ended or open ended or text$ or narrative$).mp. or (life world or life-world or conversation analys?s or personal experience$ or theoretical saturation).mp. or ((lived or life) adj experience$).mp. or cluster sampl$.mp. or observational method$.af. or content analysis.af. or (constant adj (comparative or comparison)).af. or ((discourse$ or discurs$) adj3 analys?s).tw. or	2269232

	narrative analys?s.af. or heidegger$.tw. or colaizzi$.tw. or spiegelberg$.tw. or (van adj manen$).tw. or (van adj kaam$).tw. or (merleau adj ponty$).tw. or husserl$.tw. or foucault$.tw. or (corbin$ adj2 strauss$).tw. or glaser$.tw. or interview*.tw. or case stud*.tw.	
6	4 and 5	3855
7	(("research evidence" adj5 (policy* or policies or govern* or politic*)) or (("use* of evidence" or "evidence use*" or "utili?ation of evidence" or "evidence utili?ation" or "uptake of evidence" or "evidence uptake" or "using evidence" or "utili?ing evidence") adj7 policy*) or (translat* adj3 (evidence or research or science or scientific or knowledge or findings) adj3 (policy* or policies or govern* or politic*)) or ("role of" adj3 (evidence or research or science or scientific or knowledge or findings) adj3 (policy* or policies or govern* or politic*)) or ("relation* between" adj3 (evidence or research or science or scientific or knowledge or findings) adj3 (policy* or policies or govern* or politic*)) or (apply* adj3 (evidence or research or science or scientific or knowledge or findings) adj3 (policy* or policies or govern* or politic*)) or (impact* adj2 (evidence or research or science or scientific or knowledge or findings) adj3 (policy* or policies or govern* or politic*))).tw. or ((evidence and (policymak* or policy-mak* or public policy* or public policies or health* policy* or health* policies)) or ((evidence or science or scientific or research or knowledge or findings or information) adj3 ("in policy*" or "in health* policy*" or "in policies" or "in health* policies" or "in govern*")) or ((evidence or science or scientific or research or knowledge or findings or information) adj3 ("into policy*" or "into health* policy*" or "into policies" or "into health* policies" or "into govern*")) or ((evidence or science or scientific or research or knowledge or findings or information) adj3 ("*to policy*" or "*to health* policy*" or "*to policies" or "*to health* policies" or "*to govern*")) or (("evidence based" or "evidence informed" or "research evidence" or (evidence adj2 use*) or (research adj2 use*) or (knowledge adj2 use*) or (research adj2 utili?ation) or (evidence adj2 utili?ation) or (knowledge adj2 utili?ation) or "using evidence" or "using research" or "using knowledge" or "utili?ing evidence" or "utili?ing research" or "utili?ing knowledge" or "knowledge translation" or "knowledge transfer" or "knowledge exchange" or "knowledge broker*" or "knowledge mobili?ation") and (policy* or policies or govern* or politic*)) or (researcher* adj2 (policy* or policies or govern* or politic*))).m_titl.	2427
8	6 or 7	**5822**
