# Supplementary material for: Mapping the Qualitative Evidence Base on the Use of Research Evidence in Health Policy-Making: A Systematic Review
Source: Int J Health Policy Manag. 2020 Nov 1;11(7):883–98. doi: 10.34172/ijhpm.2020.201 (PMC9808178; doi:10.34172/ijhpm.2020.201)
Supplement: Supplementary file 2 — List of Included Studies. [file ijhpm-11-883-s002.pdf]

Supplementary file 2. List of Included Studies

This file presents references for the review's 319 included studies. The list is organized alphabetically. NB the citation numbers used in this document do not correspond with citations in the text of the paper.

1. Abekah-Nkrumah G, Issiaka S, Virgil L, Ermel J. A review of the process of knowledge transfer and use of evidence in reproductive and child health in Ghana. Health Res Policy Syst. 2018;16(1):75. doi:10.1186/s12961-018-0350-9
2. Paz Aguilera X, Espinosa-Marty C, Castillo-Laborde C, Gonzalez C. From instinct to evidence: the role of data in country decision-making in Chile. Glob Health Action. 2017;10(sup1):1266176. doi:10.3402/gha.v9.32611
3. Albert MA, Fretheim A, Maïga D. Factors influencing the utilization of research findings by health policy-makers in a developing country: the selection of Mali's essential medicines. Health Res Policy Syst. 2007;5:2. doi:10.1186/1478-4505-5-2
4. llen P, Jacob RR, Lakshman M, Best LA, Bass K, Brownson RC. Lessons learned in promoting evidence-based public health: perspectives from managers in state public health departments. J Community Health. 2018;43(5):856-863. doi:10.1007/s10900-018-0494-0
5. Allen ST, Ruiz MS, O'Rourke A. The evidence does not speak for itself: the role of research evidence in shaping policy change for the implementation of publicly funded syringe exchange programs in three US cities. Int J Drug Policy. 2015;26(7):688-695. doi:10.1016/j.drugpo.2015.04.008
6. Anderson R. The policy impact of population health surveys: an illustration of the measurement challenges using the NSW Health Survey. Evid Policy. 2006;2(2):167-183. doi:10.1332/174426406777068975
7. Apollonio DE, Bero LA. Interpretation and use of evidence in state policymaking: a qualitative analysis. BMJ Open. 2017;7(2):e012738. doi:10.1136/bmjopen-2016-012738
8. Armstrong R, Waters E, Moore L, et al. Understanding evidence: a statewide survey to explore evidence-informed public health decision-making in a local government setting. Implement Sci. 2014;9:188. doi:10.1186/s13012-014-0188-7
9. Aro AR, Bertram M, Hämäläinen RM, et al. Integrating research evidence and physical activity policy making-REPOPA project. Health Promot Int. 2016;31(2):430-439. doi:10.1093/heapro/dav002
10. Atkins L, Kelly MP, Littleford C, Leng G, Michie S. Reversing the pipeline? implementing public health evidence-based guidance in english local government. Implement Sci. 2017;12(1):63. doi:10.1186/s13012-017-0589-5
11. Baghbanian A, Hughes I, Kebriaei A, Khavarpour FA. Adaptive decision-making: how Australian healthcare managers decide. Aust Health Rev. 2012;36(1):49-56. doi:10.1071/ah10971
12. Ballard M, Tran J, Hersch F, Lockwood A, Hartigan P, Montgomery P. Supporting better evidence generation and use within social innovation in health in low- and middle-income countries: a qualitative study. PLoS One. 2017;12(1):e0170367. doi:10.1371/journal.pone.0170367

13. Barnsley K, Walters EH, Wood-Baker R. Political barriers to evidence-based tobacco control policy: cronyism and cognitive dissonance, a Tasmanian case study. Evid Policy. 2017;13(2):343-364. doi:10.1332/174426416x14615120637063
14. Batchelor K, Freeman AC, Robbins A, Dudley T, Phillips N. Formative assessment of use of behavioral data in HIV prevention: Texas. AIDS Behav. 2005;9(2 Suppl):S29-40. doi:10.1007/s10461-005-3943-6
15. Baum FE, Laris P, Fisher M, Newman L, Macdougall C. "Never mind the logic, give me the numbers": former Australian health ministers' perspectives on the social determinants of health. Soc Sci Med. 2013;87:138-146. doi:10.1016/j.socscimed.2013.03.033
16. Becker LA, Loch MR, Rodrigo Siqueira R. [Barriers perceived by health directors for evidenced-based decision-making]. Rev Panam Salud Publica. 2017;41:e147. doi:10.26633/rpsp.2017.147
17. Beenstock J, Sowden S, Hunter DJ, White M. Are health and well-being strategies in England fit for purpose? a thematic content analysis. J Public Health. 2015;37(3):461-469. doi:10.1093/pubmed/fdu073
18. Behague D, Storeng K. Pragmatic politics and epistemological diversity: the contested and authoritative uses of historical evidence in the Safe Motherhood Initiative. Evid Policy. 2013;9(1):65-85. doi:10.1332/174426413x663724
19. Behague D, Tawiah C, Rosato M, Some T, Morrison J. Evidence-based policy-making: the implications of globally-applicable research for context-specific problem-solving in developing countries. Soc Sci Med. 2009;69(10):1539-1546. doi:10.1016/j.socscimed.2009.08.006
20. Bell E, Seidel BM. The evidence-policy divide: a 'critical computational linguistics' approach to the language of 18 health agency CEOs from 9 countries. BMC Public Health. 2012;12(1):932. doi:10.1186/1471-2458-12-932
21. Bennett S, Corluka A, Doherty J, et al. Influencing policy change: the experience of health think tanks in low- and middle-income countries. Health Policy Plan. 2012;27(3):194-203. doi:10.1093/heapol/czr035
22. Bennett S, Paina L, Ssengooba F, Waswa D, M'Imunya JM. The impact of Fogarty International Center research training programs on public health policy and program development in Kenya and Uganda. BMC Public Health. 2013;13:770. doi:10.1186/1471-2458-13-770
23. Bennett T, Holloway K. Is UK drug policy evidence based? Int J Drug Policy. 2010;21(5):411-417. doi:10.1016/j.drugpo.2010.02.004
24. Bertram M, Loncarevic N, Radl-Karimi C, Thøgersen M, Skovgaard T, Aro AR. Contextually tailored interventions can increase evidence-informed policy-making on health-enhancing physical activity: the experiences of two Danish municipalities. Health Res Policy Syst. 2018;16(1):14. doi:10.1186/s12961-018-0290-4
25. Bickford JJ, Kothari AR. Research and knowledge in Ontario tobacco control networks. Can J Public Health. 2008;99(4):297-300. doi:10.1007/bf03403759
26. Birkeland S, Murphy-Graham E, Weiss C. Good reasons for ignoring good evaluation: the case of the drug abuse resistance education (D.A.R.E.) program. Eval Program Plann. 2005;28(3):247-256. doi:10.1016/j.evalprogplan.2005.04.001
27. Blume S, Tump J. Evidence and policymaking: the introduction of MMR vaccine in the Netherlands. Soc Sci Med. 2010;71(6):1049-1055. doi:10.1016/j.socscimed.2010.06.023

28. Boswell J. ‘Hoisted with our own petard’: evidence and democratic deliberation on obesity. Policy Sci. 2014;47(4):345-365. doi:10.1007/s11077-014-9195-4
29. Bowen S, Erickson T, Martens PJ, Crockett S. More than "using research": the real challenges in promoting evidence-informed decision-making. Healthc Policy. 2009;4(3):87-102.
30. Bowen S, Zwi A, Sainsbury P. What evidence informs government population health policy? lessons from early childhood intervention policy in Australia. N S W Public Health Bull. 2005;16(11-12):180-184. doi:10.1071/nb05050
31. Bowen S, Zwi AB, Sainsbury P, Whitehead M. Killer facts, politics and other influences: what evidence triggered early childhood intervention policies in Australia? Evid Policy. 2009;5(1):5-32. doi:10.1332/174426409x395394
32. Brennan SE, Cumpston M, Misso ML, McDonald S, Murphy MJ, Green SE. Design and formative evaluation of the Policy Liaison Initiative: a long-term knowledge translation strategy to encourage and support the use of Cochrane systematic reviews for informing health policy. Evid Policy. 2016;12(1):25-52. doi:10.1332/174426415x14291899424526
33. Bryant T. Role of knowledge in public health and health promotion policy change. Health Promot Int. 2002;17(1):89-98. doi:10.1093/heapro/17.1.89
34. Burchett HED, Lavis JN, Mayhew SH, Dobrow MJ. Perceptions of the usefulness and use of research conducted in other countries. Evid Policy. 2012;8(1):7-16. doi:10.1332/174426412x620100
35. Burchett HED, Mayhew SH, Lavis JN, Dobrow MJ. The usefulness of different types of health research: perspectives from a low-income country. Evid Policy. 2015;11(1):19-33. doi:10.1332/174426514x13990430410723
36. Burris H, Parkhurst J, Adu-Sarkodie Y, Mayaud P. Getting research into policy - Herpes simplex virus type-2 (HSV-2) treatment and HIV infection: international guidelines formulation and the case of Ghana. Health Res Policy Syst. 2011;9(Suppl 1):S5. doi:10.1186/1478-4505-9-s1-s5
37. Cacari-Stone L, Wallerstein N, Garcia AP, Minkler M. The promise of community-based participatory research for health equity: a conceptual model for bridging evidence with policy. Am J Public Health. 2014;104(9):1615-1623. doi:10.2105/ajph.2014.301961
38. Cairney P. Evidence-based best practice is more political than it looks: a case study of the 'Scottish Approach'. Evid Policy. 2017;13(3):499-515. doi:10.1332/174426416x14609261565901
39. Cameron A, Salisbury C, Lart R, Stewart K, Peckham S, Calnan M, et al. Policy makers' perceptions on the use of evidence from evaluations. Evid Policy. 2011;7(4):429-447. doi:10.1332/174426411x603443
40. Campbell DM, Redman S, Jorm L, Cooke M, Zwi AB, Rychetnik L. Increasing the use of evidence in health policy: practice and views of policy makers and researchers. Aust New Zealand Health Policy. 2009;6:21. doi:10.1186/1743-8462-6-21
41. Carey G, Crammond B. Action on the social determinants of health: views from inside the policy process. Soc Sci Med. 2015;128:134-141. doi:10.1016/j.socscimed.2015.01.024
42. Casswell S, Stewart L, Duignan P. The negotiation of New Zealand alcohol policy in a decade of stabilized consumption and political change: the role of research. Addiction. 1993;88 Suppl:9S-17S. doi:10.1111/j.1360-0443.1993.tb02157.x

43. Castellani T, Valente A, Cori L, Bianchi F. Detecting the use of evidence in a meta-policy. Evid Policy. 2016;12(1):91-107. doi:10.1332/174426415x14430152798949
44. Cenko C, Pulvirenti M. Politics of evidence: the communication of evidence by 'stakeholders' when advocating for tobacco point-of-sale display bans in Australia. Aust J Public Adm. 2015;74(2):142-150. doi:10.1111/1467-8500.12138
45. Coburn AF. The role of health services research in developing state health policy. Health Aff (Millwood). 1998;17(1):139-151. doi:10.1377/hlthaff.17.1.139
46. Cockcroft A, Masisi M, Thabane L, Andersson N. Building capacities of elected national representatives to interpret and to use evidence for health-related policy decisions: a case study from Botswana. J Public Health Policy. 2014;35(4):475-488. doi:10.1057/jphp.2014.30
47. Colón-Ramos U, Lindsay AC, Monge-Rojas R, Greaney ML, Campos H, Peterson KE. Translating research into action: a case study on trans fatty acid research and nutrition policy in Costa Rica. Health Policy Plan. 2007;22(6):363-374. doi:10.1093/heapol/czm030
48. Craveiro I, Hortale V, Oliveira APC, Dal Poz M, Portela G, Dussault G. The utilization of research evidence in Health Workforce Policies: the perspectives of Portuguese and Brazilian National Policy-Makers. J Public Health (Oxf). 2018;40(Suppl_1):i50-i56. doi:10.1093/pubmed/fdx148
49. Dalglish SL, Rodríguez DC, Harouna A, Surkan PJ. Knowledge and power in policy-making for child survival in Niger. Soc Sci Med. 2017;177:150-157. doi:10.1016/j.socscimed.2017.01.056
50. Daniels K, Lewin S. Translating research into maternal health care policy: a qualitative case study of the use of evidence in policies for the treatment of eclampsia and pre-eclampsia in South Africa. Health Res Policy Syst. 2008;6:12. doi:10.1186/1478-4505-6-12
51. de Goede J, Putters K, van Oers H. Utilization of epidemiological research for the development of local public health policy in the Netherlands: a case study approach. Soc Sci Med. 2012;74(5):707-714. doi:10.1016/j.socscimed.2011.11.014
52. de Goede J, Steenkamer B, Treurniet H, Putters K, van Oers H. Public health knowledge utilisation by policy actors: an evaluation study in Midden-Holland, the Netherlands. Evid Policy. 2011;7(1):7-24. doi:10.1332/174426411x552972
53. de Haas B, van der Kwaak A. Exploring linkages between research, policy and practice in the Netherlands: perspectives on sexual and reproductive health and rights knowledge flows. Health Res Policy Syst. 2017;15(1):40. doi:10.1186/s12961-017-0201-0
54. Deas L, Mattu L, Gnich W. Intelligent policy making? key actors' perspectives on the development and implementation of an early years' initiative in Scotland's public health arena. Soc Sci Med. 2013;96:1-8. doi:10.1016/j.socscimed.2013.07.001
55. Dhimal M, Pandey AR, Aryal KK, et al. Utilization of health research recommendation in policy and planning in Nepal. J Nepal Health Res Counc. 2016;14(34):165-172.
56. Dobbins M, Jack S, Thomas H, Kothari A. Public health decision-makers' informational needs and preferences for receiving research evidence. Worldviews Evid Based Nurs. 2007;4(3):156-163. doi:10.1111/j.1741-6787.2007.00089.x
57. Dodson EA, Geary NA, Brownson RC. State legislators' sources and use of information: bridging the gap between research and policy. Health Educ Res. 2015;30(6):840-848. doi:10.1093/her/cyv044

58. Driedger SM, Cooper EJ, Moghadas SM. Developing model-based public health policy through knowledge translation: the need for a 'Communities of Practice'. Public Health. 2014;128(6):561-567. doi:10.1016/j.puhe.2013.10.009
59. D'Souza BJ, Parkhurst JO. When "good evidence" is not enough: a case of global malaria policy development. Glob Chall. 2018;2(9):1700077. doi:10.1002/gch2.201700077
60. Duke K. Evidence-based policy making? the interplay between research and the development of prison drugs policy. Criminol Crim Justice. 2001;1(3):277-300. doi:10.1177/1466802501001003002
61. Duke K, Thom B. The role of evidence and the expert in contemporary processes of governance: the case of opioid substitution treatment policy in England. Int J Drug Policy. 2014;25(5):964-971. doi:10.1016/j.drugpo.2014.01.015
62. DuVal G, Shah S. When does evidence from clinical trials influence health policy? a qualitative study of officials in nine African countries of the factors behind the HIV policy decision to adopt Option B+. Evid Policy. 2020;16(1):123-144. doi:10.1332/174426418x15337550449872
63. El-Jardali F, Ataya N, Jamal D, Jaafar M. A multi-faceted approach to promote knowledge translation platforms in eastern Mediterranean countries: climate for evidence-informed policy. Health Res Policy Syst. 2012;10:15. doi:10.1186/1478-4505-10-15
64. El-Jardali F, Bou-Karroum L, Ataya N, El-Ghali HA, Hammoud R. A retrospective health policy analysis of the development and implementation of the voluntary health insurance system in Lebanon: learning from failure. Soc Sci Med. 2014;123:45-54. doi:10.1016/j.socscimed.2014.10.044
65. El-Jardali F, Hammoud R, Younan L, et al. The making of nursing practice law in Lebanon: a policy analysis case study. Health Res Policy Syst. 2014;12:52. doi:10.1186/1478-4505-12-52
66. El-Jardali F, Lavis JN, Ataya N, Jamal D, Ammar W, Raouf S. Use of health systems evidence by policymakers in eastern Mediterranean countries: views, practices, and contextual influences. BMC Health Serv Res. 2012;12:200. doi:10.1186/1472-6963-12-200
67. El-Jardali F, Lavis JN, Jamal D, Ataya N, Dimassi H. Evidence-informed health policies in eastern Mediterranean countries: comparing views of policy makers and researchers. Evid Policy. 2014;10(3):397-420. doi:10.1332/174426514x672380
68. Ellen ME, Horowitz E, Vaknin S, Lavis JN. Views of health system policymakers on the role of research in health policymaking in Israel. Isr J Health Policy Res. 2016;5:24. doi:10.1186/s13584-016-0088-1
69. Elliott H, Popay J. How are policy makers using evidence? models of research utilisation and local NHS policy making. J Epidemiol Community Health. 2000;54(6):461-468. doi:10.1136/jech.54.6.461
70. Ensor T, Clapham S, Prasai DP. What drives health policy formulation: insights from the Nepal maternity incentive scheme? Health Policy. 2009;90(2-3):247-253. doi:10.1016/j.healthpol.2008.06.009
71. Eriksson L, Edman J. Knowledge, values, and needle exchange programs in Sweden. Contemp Drug Probl. 2017;44(2):105-124. doi:10.1177/0091450917700143
72. Escribano-Ferrer B, Webster J, Gyapong M. Assessing the impact of health research on health policies: a study of the Dodowa Health Research Centre, Ghana. BMC Health Serv Res. 2017;17(1):435. doi:10.1186/s12913-017-2383-0

73. Ettelt S. The politics of evidence use in health policy making in Germany-the case of regulating hospital minimum volumes. J Health Polit Policy Law. 2017;42(3):513-538. doi:10.1215/03616878-3802965
74. Ettelt S, Mays N, Nolte E. Policy learning from abroad: why it is more difficult than it seems. Policy Polit. 2012;40(4):491-504. doi:10.1332/030557312x643786
75. Evans BA, Snooks H, Howson H, Davies M. How hard can it be to include research evidence and evaluation in local health policy implementation? results from a mixed methods study. Implement Sci. 2013;8:17. doi:10.1186/1748-5908-8-17
76. Evans D. Patient and public involvement in research in the English NHS: a documentary analysis of the complex interplay of evidence and policy. Evid Policy. 2014;10(3):361-377. doi:10.1332/174426413x662770
77. Fazli GS, Creatore MI, Matheson FI, et al. Identifying mechanisms for facilitating knowledge to action strategies targeting the built environment. BMC Public Health. 2017;17(1):1. doi:10.1186/s12889-016-3954-4
78. Feldman PH, Nadash P, Gursen M. Improving communication between researchers and policy makers in long-term care: or, researchers are from Mars; policy makers are from Venus. Gerontologist. 2001;41(3):312-321. doi:10.1093/geront/41.3.312
79. Fickel JJ, Thrush CR. Policymaker use of quality of care information. Int J Qual Health Care. 2005;17(6):497-504. doi:10.1093/intqhc/mzi063
80. Field P, Gauld R, Lawrence M. Enhancing evidence use in public health nutrition policymaking: theoretical insights from a New Zealand case study. Health Res Policy Syst. 2016;14(1):84. doi:10.1186/s12961-016-0154-8
81. Flitcroft K, Gillespie J, Carter S, Salkeld G, Trevena L. Incorporating evidence and politics in health policy: can institutionalising evidence review make a difference? Evid Policy. 2014;10(3):439-455. doi:10.1332/174426514x672399
82. Flitcroft K, Gillespie J, Salkeld G, Carter S, Trevena L. Getting evidence into policy: the need for deliberative strategies? Soc Sci Med. 2011;72(7):1039-1046. doi:10.1016/j.socscimed.2011.01.034
83. Flitcroft K, Gillespie J, Carter S, Trevena L, Salkeld G. When good evidence is not enough: the role of context in bowel cancer screening policy in New Zealand. Evid Policy. 2011;7(3):307-326. doi:10.1332/174426411x591735
84. Flitcroft K, Salkeld G, Gillespie J, Trevena L, Irwig L. Fifteen years of bowel cancer screening policy in Australia: putting evidence into practice? Med J Aust. 2010;193(1):37-42. doi:10.5694/j.1326-5377.2010.tb03739.x
85. Florin D. Scientific uncertainty and the role of expert advice: the case of health checks for coronary heart disease prevention by general practitioners in the UK. Soc Sci Med. 1999;49(9):1269-1283. doi:10.1016/s0277-9536(99)00165-3
86. Florio E, Demartini JR. The use of information by policymakers at the local community level. Knowledge. 1993;15(1):106-123. doi:10.1177/107554709301500104
87. Forsetlund L, Bjørndal A. The potential for research-based information in public health: identifying unrecognised information needs. BMC Public Health. 2001;1:1. doi:10.1186/1471-2458-1-1

88. Francis D, Turley R, Thomson H, Weightman A, Waters E, Moore L. Supporting the needs of public health decision-makers and review authors in the UK. J Public Health (Oxf). 2015;37(1):172-174. doi:10.1093/pubmed/fdu089
89. Fraser A, Baeza JI, Boaz A. 'Holding the line': a qualitative study of the role of evidence in early phase decision-making in the reconfiguration of stroke services in London. Health Res Policy Syst. 2017;15(1):45. doi:10.1186/s12961-017-0207-7
90. Freebairn L, Atkinson JA, Kelly PM, McDonnell G, Rychetnik L. Decision makers' experience of participatory dynamic simulation modelling: methods for public health policy. BMC Med Inform Decis Mak. 2018;18(1):131. doi:10.1186/s12911-018-0707-6
91. Frey K. Revising road safety policy: the role of systematic evidence in Switzerland. Governance. 2010;23(4):667-690. doi:10.1111/j.1468-0491.2010.01503.x
92. Frey K, Widmer T. Revising Swiss policies: the influence of efficiency analyses. Am J Eval. 2011;32(4):494-517. doi:10.1177/1098214011401902
93. Gajjar D, Zwi AB, Hill PS, Shannon C. A case study in the use of evidence in a changing political context: an Aboriginal and Torres Strait Islander health service re-examines practice models, governance and financing. Aust Health Rev. 2014;38(4):383-386. doi:10.1071/ah13221
94. Gkeredakis E, Swan J, Powell J, et al. Mind the gap: Understanding utilisation of evidence and policy in health care management practice. J Health Organ Manag. 2011;25(3):298-314. doi:10.1108/14777261111143545
95. Gollust SE, Seymour JW, Pany MJ, Goss A, Meisel ZF, Grande D. Mutual distrust: perspectives from researchers and policy makers on the research to policy gap in 2013 and recommendations for the future. Inquiry. 2017;54:46958017705465. doi:10.1177/0046958017705465
96. Gordon EJ. The political contexts of evidence-based medicine: policymaking for daily hemodialysis. Soc Sci Med. 2006;62(11):2707-2719. doi:10.1016/j.socscimed.2005.11.024
97. Greaves DE. Evidence-based management of Caribbean health systems: barriers and opportunities. Int J Health Gov. 2017;22(2):104-117. doi:10.1108/ijhg-01-2017-0001
98. Green J. Epistemology, evidence and experience: evidence based health care in the work of Accident Alliances. Sociol Health Illn. 2000;22(4):453-476. doi:10.1111/1467-9566.00214
99. Greyson DL, Cunningham C, Morgan S. Information behaviour of Canadian pharmaceutical policy makers. Health Info Libr J. 2012;29(1):16-27. doi:10.1111/j.1471-1842.2011.00969.x
100. Guell C, Mackett R, Ogilvie D. Negotiating multisectoral evidence: a qualitative study of knowledge exchange at the intersection of transport and public health. BMC Public Health. 2017;17(1):17. doi:10.1186/s12889-016-3940-x
101. Hämäläinen RM, Aro AR, van de Goor I, et al. Exploring the use of research evidence in health-enhancing physical activity policies. Health Res Policy Syst. 2015;13:43. doi:10.1186/s12961-015-0047-2
102. Haq Z, Hafeez A, Zafar S, Ghaffar A. Dynamics of evidence-informed health policy making in Pakistan. Health Policy Plan. 2017;32(10):1449-1456. doi:10.1093/heapol/czx128
103. Harpham T, Tuan T. From research evidence to policy: mental health care in Viet Nam. Bull World Health Organ. 2006;84(8):664-668. doi:10.2471/blt.05.027789
104. Harries U, Elliott H, Higgins A. Evidence-based policy-making in the NHS: exploring the interface between research and the commissioning process. J Public Health Med. 1999;21(1):29-36. doi:10.1093/pubmed/21.1.29

105. Harris-Roxas B, Haigh F, Travaglia J, Kemp L. Evaluating the impact of equity focused health impact assessment on health service planning: three case studies. BMC Health Serv Res. 2014;14:371. doi:10.1186/1472-6963-14-371
106. Hawkes S, B KA, Jadeja N, et al. Strengthening capacity to apply health research evidence in policy making: experience from four countries. Health Policy Plan. 2016;31(2):161-170. doi:10.1093/heapol/czv032
107. Hawkins B, Alvarez Rosete A. Judicialization and health policy in Colombia: the implications for evidence-informed policymaking. Policy Stud J. 2019;47(4):953-977. doi:10.1111/psj.12230
108. Haynes AS, Derrick GE, Redman S, et al. Identifying trustworthy experts: how do policymakers find and assess public health researchers worth consulting or collaborating with? PLoS One. 2012;7(3):e32665. doi:10.1371/journal.pone.0032665
109. Haynes AS, Gillespie JA, Derrick GE, et al. Galvanizers, guides, champions, and shields: the many ways that policymakers use public health researchers. Milbank Q. 2011;89(4):564-598. doi:10.1111/j.1468-0009.2011.00643.x
110. Hennink M, Stephenson R. Using research to inform health policy: barriers and strategies in developing countries. J Health Commun. 2005;10(2):163-180. doi:10.1080/10810730590915128
111. Higashi H, Khuong TA, Ngo AD, Hill PS. Evidence and decision making: tobacco control policy and legislation in Vietnam. Int J Health Plann Manage. 2013;28(1):e72-94. doi:10.1002/hpm.2135
112. Hinchcliff R, Ivers RQ, Poulos R, Senserrick T. Utilization of research in policymaking for graduated driver licensing. Am J Public Health. 2010;100(11):2052-2058. doi:10.2105/ajph.2009.184713
113. Hivon M, Lehoux P, Denis JL, Tailliez S. Use of health technology assessment in decision making: coresponsibility of users and producers? Int J Technol Assess Health Care. 2005;21(2):268-275.
114. Hughes CE. Evidence-based policy or policy-based evidence? the role of evidence in the development and implementation of the Illicit Drug Diversion Initiative. Drug Alcohol Rev. 2007;26(4):363-368. doi:10.1080/09595230701373859
115. Hunsmann M. Limits to evidence-based health policymaking: policy hurdles to structural HIV prevention in Tanzania. Soc Sci Med. 2012;74(10):1477-1485. doi:10.1016/j.socscimed.2012.01.023
116. Hunter BM, Requejo JH, Pope I, Daelmans B, Murray SF. National health policy-makers' views on the clarity and utility of Countdown to 2015 country profiles and reports: findings from two exploratory qualitative studies. Health Res Policy Syst. 2014;12:40. doi:10.1186/1478-4505-12-40
117. Hutchinson E. The development of health policy in Malawi: the influence of context, evidence and links in the creation of a national policy for cotrimoxazole prophylaxis. Malawi Med J. 2011;23(4):109-114.
118. Hutchinson E, Parkhurst J, Phiri S, et al. National policy development for cotrimoxazole prophylaxis in Malawi, Uganda and Zambia: the relationship between context, evidence and links. Health Res Policy Syst. 2011;9(Suppl 1):S6. doi:10.1186/1478-4505-9-s1-s6

119. Hyde JK, Mackie TI, Palinkas LA, Niemi E, Leslie LK. Evidence use in mental health policy making for children in foster care. Adm Policy Ment Health. 2016;43(1):52-66. doi:10.1007/s10488-015-0633-1
120. Hyder AA, Corluka A, Winch PJ, et al. National policy-makers speak out: are researchers giving them what they need? Health Policy Plan. 2011;26(1):73-82. doi:10.1093/heapol/czq020
121. Imani-Nasab MH, Seyedin H, Majdzadeh R, Yazdizadeh B, Salehi M. Development of evidence-based health policy documents in developing countries: a case of Iran. Glob J Health Sci. 2014;6(3):27-36. doi:10.5539/gjhs.v6n3p27
122. Hasan Imani-Nasab M, Seyedin H, Yazdizadeh B, Majdzadeh R. A qualitative assessment of the evidence utilization for health policy-making on the basis of SUPPORT tools in a developing country. Int J Health Policy Manag. 2017;6(8):457-465. doi:10.15171/ijhpm.2016.158
123. Ir P, Bigdeli M, Meessen B, Van Damme W. Translating knowledge into policy and action to promote health equity: the Health Equity Fund policy process in Cambodia 2000-2008. Health Policy. 2010;96(3):200-209. doi:10.1016/j.healthpol.2010.02.003
124. Iram Y, Shaikh BT. Use of evidence for decision making: a qualitative exploratory study on MNCH program, Pakistan. Pak J Public Health. 2011;1(1):6-10.
125. Jack SM, Brooks S, Furgal CM, Dobbins M. Knowledge transfer and exchange processes for environmental health issues in Canadian Aboriginal communities. Int J Environ Res Public Health. 2010;7(2):651-674. doi:10.3390/ijerph7020651
126. Jain B, Hiligsmann M, Mathew JL, Evers SM. Analysis of a small group of stakeholders regarding advancing health technology assessment in India. Value Health Reg Issues. 2014;3:167-171. doi:10.1016/j.vhri.2014.04.006
127. Jakobsen MW, Lau CJ, Skovgaard T, Hämäläinen RM, Aro AR. Use of research evidence in policymaking in three Danish municipalities. Evid Policy. 2018;14(4):589-611. doi:10.1332/174426417x14982331542543
128. Jewell CJ, Bero LA. "Developing good taste in evidence": facilitators of and hindrances to evidence-informed health policymaking in state government. Milbank Q. 2008;86(2):177-208. doi:10.1111/j.1468-0009.2008.00519.x
129. Johns DM, Bayer R, Fairchild AL. Evidence and the politics of deimplementation: the rise and decline of the “counseling and testing” paradigm for HIV prevention at the US Centers for Disease Control and Prevention. Milbank Q. 2016;94(1):126-162. doi:10.1111/1468-0009.12183
130. Johnson SP, Greenfield TK, Giesbrecht N, Kaskutas LA, Anglin L. The role of research in the development of U.S. federal alcohol control policy. Contemp Drug Probl. 2004;31(4):737-758. doi:10.1177/009145090403100407
131. Jones DK, Louis CJ. Using evidence to inform state health policy making: lessons from four states comparing Obamacare and infant mortality. J Health Polit Policy Law. 2018;43(3):377-399. doi:10.1215/03616878-4366148
132. Jönsson K, Phoummalaysith B, Wahlström R, Tomson G. Health policy evolution in Lao People's Democratic Republic: context, processes and agency. Health Policy Plan. 2015;30(4):518-527. doi:10.1093/heapol/czu017

133. Jönsson K, Tomson G, Jönsson C, Kounnavong S, Wahlström R. Health systems research in Lao PDR: capacity development for getting research into policy and practice. Health Res Policy Syst. 2007;5:11. doi:10.1186/1478-4505-5-11
134. Jou J, Nanney MS, Walker E, Callanan R, Weisman S, Gollust SE. Using obesity research to shape obesity policy in Minnesota: stakeholder insights and feasibility of recommendations. J Public Health Manag Pract. 2018;24(3):195-203. doi:10.1097/phh.0000000000000637
135. Kapiriri L, Norheim OF, Heggenhougen K. Using burden of disease information for health planning in developing countries: the experience from Uganda. Soc Sci Med. 2003;56(12):2433-2441. doi:10.1016/s0277-9536(02)00246-0
136. Kapiriri L, Sinding C, Arnold E. How do development assistance partners conceptualise and prioritise evidence in Priority Setting (PS) for health programmes relevant to low income countries? a qualitative study. Evid Policy. 2017;13(2):255-273. doi:10.1332/174426416x14636037877986
137. Karlsson LE, Jakobsen MW, Heiberg MW, Aro AR. Involvement of external stakeholders in local health policymaking process: a case study from Odense Municipality, Denmark. Evid Policy. 2017;13(3):433-454. doi:10.1332/174426416x14609162710134
138. Katikireddi SV, Bond L, Hilton S. Perspectives on econometric modelling to inform policy: a UK qualitative case study of minimum unit pricing of alcohol. Eur J Public Health. 2014;24(3):490-495. doi:10.1093/eurpub/ckt206
139. Katikireddi SV, Hilton S, Bond L. The role of the Sheffield model on the minimum unit pricing of alcohol debate: the importance of a rhetorical perspective. Evid Policy. 2016;12(4):521-539. doi:10.1332/174426415x14430986392944
140. Katikireddi SV, Hilton S, Bonell C, Bond L. Understanding the development of minimum unit pricing of alcohol in Scotland: a qualitative study of the policy process. PLoS One. 2014;9(3):e91185. doi:10.1371/journal.pone.0091185
141. Katz A, Gajjar D, Zwi AB, Hill PS. Great expectations: an analysis of researchers' and policy makers' perceptions of the potential value of the Australian indigenous burden of disease study for policy. Int J Health Plann Manage. 2018;33(1):e181-e193. doi:10.1002/hpm.2445
142. Kelly MP, Atkins L, Littleford C, Leng G, Michie S. Evidence-based medicine meets democracy: the role of evidence-based public health guidelines in local government. J Public Health (Oxf). 2017;39(4):678-684. doi:10.1093/pubmed/fdx002
143. Kok MO, Gyapong JO, Wolffers I, Ofori-Adjei D, Ruitenberg J. Which health research gets used and why? an empirical analysis of 30 cases. Health Res Policy Syst. 2016;14(1):36. doi:10.1186/s12961-016-0107-2
144. Kothari A, Birch S, Charles C. "Interaction" and research utilisation in health policies and programs: does it work? Health Policy. 2005;71(1):117-125. doi:10.1016/j.healthpol.2004.03.010
145. Kurko T, Silvast A, Wahlroos H, Pietilä K, Airaksinen M. Is pharmaceutical policy evidence-informed? a case of the deregulation process of nicotine replacement therapy products in Finland. Health Policy. 2012;105(2-3):246-255. doi:10.1016/j.healthpol.2012.02.013
146. La Brooy C, Kelaher M. The research-policy-deliberation nexus: a case study approach. Health Res Policy Syst. 2017;15(1):75. doi:10.1186/s12961-017-0239-z

147. Lafi R, Robinson S, Williams I. Economic evaluation and the Jordan Rational Drug List: an exploratory study of national-level priority setting. Value Health. 2012;15(5):771-776. doi:10.1016/j.jval.2012.04.006
148. Lairumbi GM, Molyneux S, Snow RW, Marsh K, Peshu N, English M. Promoting the social value of research in Kenya: examining the practical aspects of collaborative partnerships using an ethical framework. Soc Sci Med. 2008;67(5):734-747. doi:10.1016/j.socscimed.2008.02.016
149. Lancaster K. Performing the evidence-based drug policy paradigm. Contemp Drug Probl. 2016;43(2):142-153. doi:10.1177/0091450916633306
150. Lancaster K, Treloar C, Ritter A. 'Naloxone works': the politics of knowledge in 'evidence-based' drug policy. Health (London). 2017;21(3):278-294. doi:10.1177/1363459316688520
151. Lavis J, Davies H, Oxman A, Denis JL, Golden-Biddle K, Ferlie E. Towards systematic reviews that inform health care management and policy-making. J Health Serv Res Policy. 2005;10 Suppl 1:35-48. doi:10.1258/1355819054308549
152. Lavis JN, Farrant MS, Stoddart GL. Barriers to employment-related healthy public policy in Canada. Health Promot Int. 2001;16(1):9-20. doi:10.1093/heapro/16.1.9
153. Lavis JN, Ross SE, Hurley JE, et al. Examining the role of health services research in public policymaking. Milbank Q. 2002;80(1):125-154. doi:10.1111/1468-0009.00005
154. Laws R, King L, Hardy LL, et al. Utilization of a population health survey in policy and practice: a case study. Health Res Policy Syst. 2013;11:4. doi:10.1186/1478-4505-11-4
155. Leslie LK, Maciolek S, Biebel K, Debordes-Jackson G, Nicholson J. Exploring knowledge exchange at the research-policy-practice interface in children's behavioral health services. Adm Policy Ment Health. 2014;41(6):822-834. doi:10.1007/s10488-014-0535-7
156. Lillefjell M, Knudtsen MS, Wist G, Ihlebæk C. From knowledge to action in public health management: experiences from a Norwegian context. Scand J Public Health. 2013;41(8):771-777. doi:10.1177/1403494813496600
157. Liu H, Muhunthan J, Ananthapavan J, Hawe P, Shiell A, Jan S. Exploring the use of economic evidence to inform investment in disease prevention - a qualitative study. Aust N Z J Public Health. 2018;42(2):200-206. doi:10.1111/1753-6405.12748
158. Liverani M, Chheng K, Parkhurst J. The making of evidence-informed health policy in Cambodia: knowledge, institutions and processes. BMJ Glob Health. 2018;3(3):e000652. doi:10.1136/bmjgh-2017-000652
159. Lomas J, Brown AD. Research and advice giving: a functional view of evidence-informed policy advice in a Canadian Ministry of Health. Milbank Q. 2009;87(4):903-926. doi:10.1111/j.1468-0009.2009.00583.x
160. MacGregor S. The impact of research on policy in the drugs field. Methodological Innovations Online Method Innov. 2011;6(1):41-57. doi:10.4256/mio.2010.0027
161. Mackenzie M, Blamey A, Hanlon P. Using and generating evidence: policy makers' reflections on commissioning and learning from the Scottish Health Demonstration Projects. Evid Policy. 2006;2(2):211-226. doi:10.1332/174426406777068885
162. Mackie TI, Sheldrick RC, Hyde J, Leslie LK. Exploring the integration of systems and social sciences to study evidence use among child welfare policy-makers. Child Welfare. 2015;94(3):33-58.

163. MacKillop E, Sheard S. The politics of health policy knowledge transfer: the evolution of the role of British health economics academic units. Evid Policy. 2019;15(4):489-507. doi:10.1332/174426418x15378681544353
164. Majdzadeh R, Yazdizadeh B, Nedjat S, Gholami J, Ahghari S. Strengthening evidence-based decision-making: is it possible without improving health system stewardship? Health Policy Plan. 2012;27(6):499-504. doi:10.1093/heapol/czr072
165. Makkar SR, Brennan S, Turner T, Williamson A, Redman S, Green S. The development of SAGE: a tool to evaluate how policymakers' engage with and use research in health policymaking. Res Eval. 2016;25(3):315-328. doi:10.1093/reseval/rvv044
166. Martin G, Currie G, Lockett A. Prospects for knowledge exchange in health policy and management: institutional and epistemic boundaries. J Health Serv Res Policy. 2011;16(4):211-217. doi:10.1258/jhsrp.2011.010132
167. May C. Mobilising modern facts: health technology assessment and the politics of evidence. Sociol Health Illn. 2006;28(5):513-532. doi:10.1111/j.1467-9566.2006.00505.x
168. Mbachu CO, Onwujekwe O, Chikezie I, Ezumah N, Das M, Uzochukwu BS. Analysing key influences over actors' use of evidence in developing policies and strategies in Nigeria: a retrospective study of the Integrated Maternal Newborn and Child Health strategy. Health Res Policy Syst. 2016;14:27. doi:10.1186/s12961-016-0098-z
169. McBride T, Coburn A, Mackinney C, Mueller K, Slifkin R, Wakefield M. Bridging health research and policy: effective dissemination strategies. J Public Health Manag Pract. 2008;14(2):150-154. doi:10.1097/01.PHH.0000311893.80701.7a
170. McGill E, Egan M, Petticrew M, et al. Trading quality for relevance: non-health decision-makers' use of evidence on the social determinants of health. BMJ Open. 2015;5(4):e007053. doi:10.1136/bmjopen-2014-007053
171. McGinty EE, Siddiqi S, Linden S, Horwitz J, Frattaroli S. Improving the use of evidence in public health policy development, enactment and implementation: a multiple-case study. Health Educ Res. 2019;34(2):129-144. doi:10.1093/her/cyy050
172. McKee M, Fulop N, Bouvier P, et al. Preventing sudden infant deaths--the slow diffusion of an idea. Health Policy. 1996;37(2):117-135. doi:10.1016/0168-8510(95)00829-2
173. Mele V, Compagni A, Cavazza M. Governing through evidence: a study of technological innovation in health care. J Public Adm Res Theory. 2014;24(4):843-877. doi:10.1093/jopart/mut016
174. Mercer SL, Sleet DA, Elder RW, Cole KH, Shults RA, Nichols JL. Translating evidence into policy: lessons learned from the case of lowering the legal blood alcohol limit for drivers. Ann Epidemiol. 2010;20(6):412-420. doi:10.1016/j.annepidem.2010.03.005
175. Mihalicza P, Leys M, Borbás I, Szigeti S, Biermann O, Kuchenmüller T. Qualitative assessment of opportunities and challenges to improve evidence-informed health policy-making in Hungary - an EVIPNet situation analysis pilot. Health Res Policy Syst. 2018;16(1):50. doi:10.1186/s12961-018-0331-z
176. Mijumbi-Deve R, Sewankambo NK. A process evaluation to assess contextual factors associated with the uptake of a rapid response service to support health systems' decision-making in Uganda. Int J Health Policy Manag. 2017;6(10):561-571. doi:10.15171/ijhpm.2017.04

177. Milat AJ, King L, Newson R, et al. Increasing the scale and adoption of population health interventions: experiences and perspectives of policy makers, practitioners, and researchers. Health Res Policy Syst. 2014;12:18. doi:10.1186/1478-4505-12-18
178. Minkler M, Garcia AP, Williams J, LoPresti T, Lilly J. Sí se puede: using participatory research to promote environmental justice in a Latino community in San Diego, California. J Urban Health. 2010;87(5):796-812. doi:10.1007/s11524-010-9490-0
179. Mirzoev T, Das M, Ebenso B, et al. Contextual influences on the role of evidence in health policy development: what can we learn from six policies in India and Nigeria? Evid Policy. 2017;13(1):59-79. doi:10.1332/174426415x14454407579925
180. Mirzoev T, Green A, Gerein N, et al. Role of evidence in maternal health policy processes in Vietnam, India and China: findings from the HEPVIC project. Evid Policy. 2013;9(4):493-511. doi:10.1332/174426413x669845
181. Mitton C, Macnab YC, Smith N, Foster L. Injury data in British Columbia: policy maker perspectives on knowledge transfer. Chronic Dis Can. 2009;29(2):70-79.
182. Mitton C, Patten S. Evidence-based priority-setting: what do the decision-makers think? J Health Serv Res Policy. 2004;9(3):146-152. doi:10.1258/1355819041403240
183. Mohtasham F, Yazdizadeh B, Zali Z, Majdzadeh R, Nedjat S. Health technology assessment in Iran: barriers and solutions. Med J Islam Repub Iran. 2016;30:321.
184. Monaghan M. Appreciating cannabis: the paradox of evidence in evidence-based policy making. Evid Policy. 2008;4(2):209-331. doi:10.1332/174426408784614662
185. Monaghan M. The complexity of evidence: reflections on research utilisation in a heavily politicised policy area. Soc Policy Soc. 2010;9(1):1-12. doi:10.1017/s1474746409990157
186. Mori AT, Kaale EA, Ngalesoni F, Norheim OF, Robberstad B. The role of evidence in the decision-making process of selecting essential medicines in developing countries: the case of Tanzania. PLoS One. 2014;9(1):e84824. doi:10.1371/journal.pone.0084824
187. Morton S. Creating research impact: the roles of research users in interactive research mobilisation. Evid Policy. 2015;11(1):35-55. doi:10.1332/174426514x13976529631798
188. Mosquera J, Gómez OL, Méndez F. Use of research results in public health in the Valle del Cauca Secretariat of Health, Colombia. Colomb Med. 2005;36(1):16-22.
189. Mubyazi GM, Gonzalez-Block MA. Research influence on antimalarial drug policy change in Tanzania: case study of replacing chloroquine with sulfadoxine-pyrimethamine as the first-line drug. Malar J. 2005;4:51. doi:10.1186/1475-2875-4-51
190. Mulvale G, McRae SA, Milicic S. Teasing apart "the tangled web" of influence of policy dialogues: lessons from a case study of dialogues about healthcare reform options for Canada. Implement Sci. 2017;12(1):96. doi:10.1186/s13012-017-0627-3
191. Mwendera C, de Jager C, Longwe H, Phiri K, Hongoro C, Mutero CM. Malaria research and its influence on anti-malarial drug policy in Malawi: a case study. Health Res Policy Syst. 2016;14(1):41. doi:10.1186/s12961-016-0108-1
192. Mwendera CA, de Jager C, Longwe H, Phiri K, Hongoro C, Mutero CM. Facilitating factors and barriers to malaria research utilization for policy development in Malawi. Malar J. 2016;15(1):512. doi:10.1186/s12936-016-1547-4
193. Mwendera CA, de Jager C, Longwe H, Phiri K, Hongoro C, Mutero CM. Changing the policy for intermittent preventive treatment with sulfadoxine-pyrimethamine during pregnancy in Malawi. Malar J. 2017;16(1):84. doi:10.1186/s12936-017-1736-9

194. Nabyonga-Orem J, Marchal B, Mafigiri D, et al. Perspectives on the role of stakeholders in knowledge translation in health policy development in Uganda. BMC Health Serv Res. 2013;13:324. doi:10.1186/1472-6963-13-324
195. Nabyonga-Orem J, Mafigiri DK, Marchal B, Ssengooba F, Macq J, Criel B. Research, evidence and policymaking: the perspectives of policy actors on improving uptake of evidence in health policy development and implementation in Uganda. BMC Public Health. 2012;12:109. doi:10.1186/1471-2458-12-109
196. Nabyonga-Orem J, Mafigiri DK, Nabudere H, Criel B. Improving knowledge translation in Uganda: more needs to be done. Pan Afr Med J. 2014;17(Suppl 1):14. doi:10.11694/pamj.supp.2014.17.1.3482
197. Nabyonga-Orem J, Mijumbi R. Evidence for informing health policy development in Low-income Countries (LICs): perspectives of policy actors in Uganda. Int J Health Policy Manag. 2015;4(5):285-293. doi:10.15171/ijhpm.2015.52
198. Nabyonga-Orem J, Nanyunja M, Marchal B, Criel B, Ssengooba F. The roles and influence of actors in the uptake of evidence: the case of malaria treatment policy change in Uganda. Implement Sci. 2014;9:150. doi:10.1186/s13012-014-0150-8
199. Nabyonga-Orem J, Ssengooba F, Macq J, Criel B. Malaria treatment policy change in Uganda: what role did evidence play? Malar J. 2014;13:345. doi:10.1186/1475-2875-13-345
200. Nabyonga-Orem J, Ssengooba F, Mijumbi R, Tashobya CK, Marchal B, Criel B. Uptake of evidence in policy development: the case of user fees for health care in public health facilities in Uganda. BMC Health Serv Res. 2014;14:639. doi:10.1186/s12913-014-0639-5
201. Nakkash RT, Torossian L, El Hajj T, Khalil J, Afifi RA. The passage of tobacco control law 174 in Lebanon: reflections on the problem, policies and politics. Health Policy Plan. 2018;33(5):633-644. doi:10.1093/heapol/czy023
202. Narain KDC, Zimmerman FJ, Richards J, et al. Evidentiary needs of US public health departments with a mission to advance equity and health: a qualitative analysis. BMJ Open. 2018;8(9):e022033. doi:10.1136/bmjopen-2018-022033
203. Nathan SA, Develin E, Grove N, Zwi AB. An Australian childhood obesity summit: the role of data and evidence in 'public' policy making. Aust New Zealand Health Policy. 2005;2:17. doi:10.1186/1743-8462-2-17
204. Naude CE, Zani B, Ongolo-Zogo P, et al. Research evidence and policy: qualitative study in selected provinces in South Africa and Cameroon. Implement Sci. 2015;10:126. doi:10.1186/s13012-015-0315-0
205. Nolan LB, Lucas R, Choi Y, Fabic MS, Adetunji JA. The contribution of demographic and health survey data to population and health policymaking: evidence from three developing countries. Afr Popul Stud. 2017;31(1):3395-3407. doi:10.11564/31-1-998
206. Nutley S, Walter I, Bland N. The institutional arrangements for connecting evidence and policy: the case of drug misuse. Public Policy Adm. 2002;17(3):76-94. doi:10.1177/095207670201700306
207. O'Donoughue Jenkins L, Kelly PM, Cherbuin N, Anstey KJ. Evaluating and using observational evidence: the contrasting views of policy makers and epidemiologists. Front Public Health. 2016;4:267. doi:10.3389/fpubh.2016.00267

208. de Oliveira APC, Dal Poz MR, Craveiro I, Gabriel M, Dussault G. Factors that influence human resources for health policy formulation: a multiple case study in Brazil and Portugal. Cad Saude Publica. 2018;34(2):e00220416. doi:10.1590/0102-311x00220416
209. O'Mullane M, Quinlivan A. Health Impact Assessment (HIA) in Ireland and the role of local government. Environ Impact Assess Rev. 2012;32(1):181-186. doi:10.1016/j.eiar.2011.08.004
210. Ongolo-Zogo P, Lavis JN, Tomson G, Sewankambo NK. Climate for evidence informed health system policymaking in Cameroon and Uganda before and after the introduction of knowledge translation platforms: a structured review of governmental policy documents. Health Res Policy Syst. 2015;13:2. doi:10.1186/1478-4505-13-2
211. Ongolo-Zogo P, Lavis JN, Tomson G, Sewankambo NK. Assessing the influence of knowledge translation platforms on health system policy processes to achieve the health millennium development goals in Cameroon and Uganda: a comparative case study. Health Policy Plan. 2018;33(4):539-554. doi:10.1093/heapol/czx194
212. Onwujekwe O, Uguru N, Russo G, et al. Role and use of evidence in policymaking: an analysis of case studies from the health sector in Nigeria. Health Res Policy Syst. 2015;13:46. doi:10.1186/s12961-015-0049-0
213. Oronje RN, Zulu EM. Contribution of a network of parliamentary committees of health to the ecosystem of evidence use in African parliaments. Evid Policy. 2018;14(3):523-535. doi:10.1332/174426418x15314037224599
214. Ottoson JM, Green LW, Beery WL, et al. Policy-contribution assessment and field-building analysis of the Robert Wood Johnson Foundation's Active Living Research Program. Am J Prev Med. 2009;36(2 Suppl):S34-43. doi:10.1016/j.amepre.2008.10.010
215. Oxman AD, Lavis JN, Fretheim A. Use of evidence in WHO recommendations. Lancet. 2007;369(9576):1883-1889. doi:10.1016/s0140-6736(07)60675-8
216. Ozawa S, Privor-Dumm LA, Nanni A, et al. Evidence-to-policy gap on hepatitis A vaccine adoption in 6 countries: Literature vs. policymakers' beliefs. Vaccine. 2014;32(32):4089-4096. doi:10.1016/j.vaccine.2014.05.026
217. Parker LE, Ritchie MJ, Kirchner JE, Owen RR. Balancing health care evidence and art to meet clinical needs: policymakers' perspectives. J Eval Clin Pract. 2009;15(6):970-975. doi:10.1111/j.1365-2753.2009.01209.x
218. Parkhurst JO. Framing, ideology and evidence: Uganda's HIV success and the development of PEPFAR's' ABC'policy for HIV prevention. Evid Policy. 2012;8(1):17-36. doi:10.1332/174426412x620119
219. Parkhurst JO, Hyde A, South A, Brehmer L, Miller A, Newell JN. Improving communication of research findings: Identifying the sources of information most important to national disease control officers in low-and middle-income countries. Trop Med Int Health. 2010;15(10):1252-1255. doi:10.1111/j.1365-3156.2010.02599.x
220. Peirson L, Ciliska D, Dobbins M, Mowat D. Building capacity for evidence informed decision making in public health: a case study of organizational change. BMC Public Health. 2012;12:137. doi:10.1186/1471-2458-12-137
221. Petrosino A, Birkeland S, Hacsi TA, Murphy-Graham E, Weiss CH. US state government and DARE: the story in four states. Evid Policy. 2006;2(3):291-319. doi:10.1332/174426406778023685

222. Petticrew M, Platt S, McCollam A, Wilson S, Thomas S. "We're not short of people telling us what the problems are. We're short of people telling us what to do": an appraisal of public policy and mental health. BMC Public Health. 2008;8:314. doi:10.1186/1471-2458-8-314
223. Petticrew M, Whitehead M, Macintyre SJ, Graham H, Egan M. Evidence for public health policy on inequalities: 1: the reality according to policymakers. J Epidemiol Community Health. 2004;58(10):811-816. doi:10.1136/jech.2003.015289
224. Phillips G, Green J. Working for the public health: politics, localism and epistemologies of practice. Sociol Health Illn. 2015;37(4):491-505. doi:10.1111/1467-9566.12214
225. Philpott A, Maher D, Grosskurth H. Translating HIV/AIDS research findings into policy: lessons from a case study of 'the Mwanza trial'. Health Policy Plan. 2002;17(2):196-201. doi:10.1093/heapol/17.2.196
226. Prinja S, Gupta R, Sharma A, Dalpath SK, Phogat A. Engaging actors for integrating health policy and systems research into policy making: case study from Haryana state in India. Indian J Community Health. 2017;29(3):320-322.
227. Probandari A, Widjanarko B, Mahendradhata Y, et al. The path to impact of operational research on tuberculosis control policies and practices in Indonesia. Glob Health Action. 2016;9:29866. doi:10.3402/gha.v9.29866
228. Purtle J, Peters R, Kolker J, Diez Roux AV. Uses of population health rankings in local policy contexts: a multisite case study. Med Care Res Rev. 2019;76(4):478-496. doi:10.1177/1077558717726115
229. Qureshi K. It's not just pills and potions? depoliticising health inequalities policy in England. Anthropol Med. 2013;20(1):1-12. doi:10.1080/13648470.2012.747593
230. Randall N. Drug policy and rationality: an exploration of the research–policy interface in Ireland. Drugs. 2011;18(4):285-294. doi:10.3109/09687637.2010.493540
231. Reddy KS, Sahay S. Voices of decision makers on evidence-based policy: a case of evolving TB/HIV co-infection policy in India. AIDS Care. 2016;28(3):397-400. doi:10.1080/09540121.2015.1096889
232. Reid G, Connolly J, Halliday W, Love AM, Higgins M, MacGregor A. Minding the gap: the barriers and facilitators of getting evidence into policy when using a knowledge-brokering approach. Evid Policy. 2017;13(1):29-38. doi:10.1332/174426416x14526131924179
233. Rispel LC, Doherty J. Research in support of health systems transformation in South Africa: the experience of the Centre for Health Policy. J Public Health Policy. 2011;32 Suppl 1:S10-29. doi:10.1057/jphp.2011.33
234. Ritter A. How do drug policy makers access research evidence? Int J Drug Policy. 2009;20(1):70-75. doi:10.1016/j.drugpo.2007.11.017
235. Roberts H, Petticrew M, Liabo K, Macintyre S. 'The Anglo-Saxon disease': a pilot study of the barriers to and facilitators of the use of randomised controlled trials of social programmes in an international context. J Epidemiol Community Health. 2012;66(11):1025-1029. doi:10.1136/jech-2011-200313
236. Rodríguez DC, Shearer J, Mariano AR, Juma PA, Dalglish SL, Bennett S. Evidence-informed policymaking in practice: country-level examples of use of evidence for iCCM policy. Health Policy Plan. 2015;30 Suppl 2:ii36-ii45. doi:10.1093/heapol/czv033
237. Roseboom KJ, van Dongen JM, Tompa E, van Tulder MW, Bosmans JE. Economic evaluations of health technologies in Dutch healthcare decision-making: a qualitative study of

the current and potential use, barriers, and facilitators. BMC Health Serv Res. 2017;17(1):89. doi:10.1186/s12913-017-1986-9
238. Rosella LC, Wilson K, Crowcroft NS, et al. Pandemic H1N1 in Canada and the use of evidence in developing public health policies--a policy analysis. Soc Sci Med. 2013;83:1-9. doi:10.1016/j.socscimed.2013.02.009
239. Ross J. The use of economic evaluation in health care: Australian decision makers' perceptions. Health Policy. 1995;31(2):103-110. doi:10.1016/0168-8510(94)00671-7
240. Rossow I, Ugland T, Baklien B. Use of research in local alcohol policy-making. Drugs Alcohol Today. 2015;15(4):192-202. doi:10.1108/dat-05-2015-0022
241. Saidi T, Salie F, Douglas TS. Towards understanding the drivers of policy change: a case study of infection control policies for multi-drug resistant tuberculosis in South Africa. Health Res Policy Syst. 2017;15(1):41. doi:10.1186/s12961-017-0203-y
242. Salter V, Enterline PE. The utilization of research in the national health policy debate: a case example. Health Policy Q. 1982;2(1):53-64.
243. Sandberg J, Persson B, Garpenby P. The dilemma of knowledge use in political decision-making: National Guidelines in a Swedish priority-setting context. Health Econ Policy Law. 2019;14(4):425-442. doi:10.1017/s1744133118000233
244. Sanders T, Grove A, Salway S, Hampshaw S, Goyder E. Incorporation of a health economic modelling tool into public health commissioning: evidence use in a politicised context. Soc Sci Med. 2017;186:122-129. doi:10.1016/j.socscimed.2017.06.011
245. Sato H, Frantz JE. Termination of the leprosy isolation policy in the US and Japan: science, policy changes, and the garbage can model. BMC Int Health Hum Rights. 2005;5(1):3. doi:10.1186/1472-698x-5-3
246. Huckel Schneider C, Campbell D, Milat A, Haynes A, Quinn E. What are the key organisational capabilities that facilitate research use in public health policy? Public Health Res Pract. 2014;25(1). doi:10.17061/phrp2511406
247. Schwartz R, Rosen B. The politics of evidence-based health policy-making. Public Money Manag. 2004;24(2):121-127. doi:10.1111/j.1467-9302.2004.00404.x
248. Shroff Z, Aulakh B, Gilson L, Agyepong IA, El-Jardali F, Ghaffar A. Incorporating research evidence into decision-making processes: researcher and decision-maker perceptions from five low- and middle-income countries. Health Res Policy Syst. 2015;13:70. doi:10.1186/s12961-015-0059-y
249. Smith KE. Institutional filters: the translation and re-circulation of ideas about health inequalities within policy. Policy Polit. 2013;41(1):81-100. doi:10.1332/030557312x655413
250. Smith KE. Health inequalities in Scotland and England: the contrasting journeys of ideas from research into policy. Soc Sci Med. 2007;64(7):1438-1449. doi:10.1016/j.socscimed.2006.11.008
251. Smith KE. The politics of ideas: the complex interplay of health inequalities research and policy. Sci Public Policy. 2014;41(5):561-574. doi:10.1093/scipol/sct085
252. Smith KE, Joyce KE. Capturing complex realities: understanding efforts to achieve evidence-based policy and practice in public health. Evid Policy. 2012;8(1):57-78. doi:10.1332/174426412x6201371

253. Smith KE, Weishaar H. Networks, advocacy and evidence in public health policymaking: insights from case studies of European Union smoke-free and English health inequalities policy debates. Evid Policy. 2018;14(3):403-430. doi:10.1332/174426418x15299596208647
254. Sosnowy CD, Weiss LJ, Maylahn CM, Pirani SJ, Katagiri NJ. Factors affecting evidence-based decision making in local health departments. Am J Prev Med. 2013;45(6):763-768. doi:10.1016/j.amepre.2013.08.004
255. Soumerai SB, Ross-Degnan D, Fortess EE, Walser BL. Determinants of change in Medicaid pharmaceutical cost sharing: does evidence affect policy? Milbank Q. 1997;75(1):11-34. doi:10.1111/1468-0009.00043
256. Ssengooba F, Atuyambe L, Kiwanuka SN, Puvanachandra P, Glass N, Hyder AA. Research translation to inform national health policies: learning from multiple perspectives in Uganda. BMC Int Health Hum Rights. 2011;11(Suppl 1):S13. doi:10.1186/1472-698x-11-s1-s13
257. Stewart E, Smith KE. 'Black magic'and'gold dust': the epistemic and political uses of evidence tools in public health policy making. Evid Policy. 2015;11(3):415-437. doi:10.1332/174426415x14381786400158
258. Strehlenert H, Richter-Sundberg L, Nyström ME, Hasson H. Evidence-informed policy formulation and implementation: a comparative case study of two national policies for improving health and social care in Sweden. Implement Sci. 2015;10:169. doi:10.1186/s13012-015-0359-1
259. Sumner A, Harpham T. The market for 'evidence' in policy processes: the case of child health policy in Andhra Pradesh, India and Viet Nam. Eur J Dev Res. 2008;20(4):712-732. doi:10.1080/09578810802493358
260. Suter E, Armitage GD. Use of a knowledge synthesis by decision makers and planners to facilitate system level integration in a large Canadian provincial health authority. Int J Integr Care. 2011;11:e011. doi:10.5334/ijic.576
261. Taylor-Robinson D, Milton B, Lloyd-Williams F, O'Flaherty M, Capewell S. Policy-makers' attitudes to decision support models for coronary heart disease: a qualitative study. J Health Serv Res Policy. 2008;13(4):209-214. doi:10.1258/jhsrp.2008.008045
262. Teerawattananon Y, Russell S. A difficult balancing act: policy actors' perspectives on using economic evaluation to inform health-care coverage decisions under the Universal Health Insurance Coverage scheme in Thailand. Value Health. 2008;11 Suppl 1:S52-60. doi:10.1111/j.1524-4733.2008.00367.x
263. Teerawattananon Y, Tantivess S, Yamabhai I, et al. The influence of cost-per-DALY information in health prioritisation and desirable features for a registry: a survey of health policy experts in Vietnam, India and Bangladesh. Health Res Policy Syst. 2016;14(1):86. doi:10.1186/s12961-016-0156-6
264. Tesfazghi K, Hill J, Jones C, Ranson H, Worrall E. National malaria vector control policy: an analysis of the decision to scale-up larviciding in Nigeria. Health Policy Plan. 2016;31(1):91-101. doi:10.1093/heapol/czv055
265. Thomson G, Wilson N, Howden-Chapman P. The use and misuse of health research by parliamentary politicians during the development of a national smokefree law. Aust New Zealand Health Policy. 2007;4:24. doi:10.1186/1743-8462-4-24

266. Tieberghien J. The role of the media in the science-policy nexus. Some critical reflections based on an analysis of the Belgian drug policy debate (1996-2003). Int J Drug Policy. 2014;25(2):276-281. doi:10.1016/j.drugpo.2013.05.014
267. Tieberghien J. Challenges and opportunities of 'good governance' for drug policy: the case of the development of Belgian drug policy between 1996 and 2003. Drugs. 2018;25(2):156-163. doi:10.1080/09687637.2016.1230594
268. Tieberghien J, Decorte T. Understanding the science–policy nexus in Belgium: an analysis of the drug policy debate (1996–2003). Drugs. 2013;20(3):241-248. doi:10.3109/09687637.2012.759904
269. Tieberghien J, Monaghan M. Public scholarship and the evidence movement: understanding and learning from Belgian drug policy development. Eur J Criminol. 2018;15(3):278-295. doi:10.1177/1477370817731413
270. Toner P, Lloyd C, Thom B, et al. Perceptions on the role of evidence: an English alcohol policy case study. Evid Policy. 2014;10(1):93-112. doi:10.1332/10.1332/174426514x13899745453819
271. Tran NT, Bennett SC, Bishnu R, Singh S. Analyzing the sources and nature of influence: how the Avahan program used evidence to influence HIV/AIDS prevention policy in India. Implement Sci. 2013;8:44. doi:10.1186/1748-5908-8-44
272. Trostle J, Bronfman M, Langer A. How do researchers influence decision-makers? case studies of Mexican policies. Health Policy Plan. 1999;14(2):103-114. doi:10.1093/heapol/14.2.103
273. Udovyk O. Models of science-policy interaction: exploring approaches to Bisphenol A management in the EU. Sci Total Environ. 2014;485-486:23-30. doi:10.1016/j.scitotenv.2014.03.046
274. Uneke CJ, Ezeoha AE, Ndukwe CD, Oyibo PG, Onwe F. Development of health policy and systems research in Nigeria: lessons for developing countries' evidence-based health policy making process and practice. Healthc Policy. 2010;6(1):e109-126.
275. Uneke CJ, Ezeoha AE, Ndukwe CD, Oyibo PG, Onwe F. Promotion of evidence-informed health policymaking in Nigeria: bridging the gap between researchers and policymakers. Glob Public Health. 2012;7(7):750-765. doi:10.1080/17441692.2012.666255
276. Uneke CJ, Ndukwe CD, Ezeoha AA, Uro-Chukwu HC, Ezeonu CT. Implementation of a health policy advisory committee as a knowledge translation platform: the Nigeria experience. Int J Health Policy Manag. 2015;4(3):161-168. doi:10.15171/ijhpm.2015.21
277. Uneke CJ, Sombie I, Keita N, Lokossou V, Johnson E, Ongolo-Zogo P. Improving maternal and child health policymaking processes in Nigeria: an assessment of policymakers' needs, barriers and facilitators of evidence-informed policymaking. Health Res Policy Syst. 2017;15(Suppl 1):48. doi:10.1186/s12961-017-0217-5
278. Utens CM, Dirksen CD, van der Weijden T, Joore MA. How to integrate research evidence on patient preferences in pharmaceutical coverage decisions and clinical practice guidelines: a qualitative study among Dutch stakeholders. Health Policy. 2016;120(1):120-128. doi:10.1016/j.healthpol.2015.10.005
279. Uzochukwu B, Onwujekwe O, Mbachu C, et al. The challenge of bridging the gap between researchers and policy makers: experiences of a Health Policy Research Group in engaging policy makers to support evidence informed policy making in Nigeria. Global Health. 2016;12(1):67. doi:10.1186/s12992-016-0209-1

280. Valente A, Castellani T, Larsen M, Aro AR. Models and visions of science–policy interaction: remarks from a Delphi study in Italy. Sci Public Policy. 2015;42(2):228-241. doi:10.1093/scipol/scu039
281. Vallgårda S. The Danish trans-fatty acids ban: alliances, mental maps and co-production of policies and research. Evid Policy. 2018;14(2):221-234. doi:10.1332/174426417x14860575527257
282. van de Goor I, Hämäläinen RM, Syed A, et al. Determinants of evidence use in public health policy making: results from a study across six EU countries. Health Policy. 2017;121(3):273-281. doi:10.1016/j.healthpol.2017.01.003
283. van den Heuvel WJ, Wieringh R, van den Heuvel LP. Utilisation of medical technology assessment in health policy. Health Policy. 1997;42(3):211-222. doi:10.1016/s0168-8510(97)00073-0
284. van der Graaf P, Forrest LF, Adams J, Shucksmith J, White M. How do public health professionals view and engage with research? a qualitative interview study and stakeholder workshop engaging public health professionals and researchers. BMC Public Health. 2017;17(1):892. doi:10.1186/s12889-017-4896-1
285. van der Putten IM, Paulus ATG, Hiligsmann M, Hutubessy RCW, Evers S. Evidence-informed vaccine decision making: the introduction of Human Papilloma Virus (HPV) vaccination in the Netherlands. Health Policy. 2019;123(3):260-266. doi:10.1016/j.healthpol.2018.09.001
286. van Egmond S, Bekker M, Bal R, van der Grinten T. Connecting evidence and policy: bringing researchers and policy makers together for effective evidence-based health policy in the Netherlands: a case study. Evid Policy. 2011;7(1):25-39. doi:10.1332/174426411x552981
287. Van Herck P, Annemans L, Sermeus W, Ramaekers D. Evidence-based health care policy in reimbursement decisions: lessons from a series of six equivocal case-studies. PLoS One. 2013;8(10):e78662. doi:10.1371/journal.pone.0078662
288. van Overveld PJM, Hermans LM, Verliefde ARD. The use of technical knowledge in European water policy-making. Environ Policy Gov. 2010;20(5):322-335. doi:10.1002/eet.546
289. van Toorn G, Dowse L. Policy claims and problem frames: a cross-case comparison of evidence-based policy in an Australian context. Evid Policy. 2016;12(1):9-24. doi:10.1332/174426415x14253873124330
290. Vargas E, Becerril-Montekio V, Gonzalez-Block M, et al. Mapping the use of research to support strategies tackling maternal and child health inequities: evidence from six countries in Africa and Latin America. Health Res Policy Syst. 2016;14:1. doi:10.1186/s12961-015-0072-1
291. Vecchione E, Parkhurst J. The use of evidence within policy evaluation in health in Ghana: implications for accountability and democratic governance. European Policy Analysis. 2015;1(2):111-131. doi:10.18278/epa.1.2.6
292. Vujcich D, Rayner M, Allender S, Fitzpatrick R. When there is not enough evidence and when evidence is not enough: an Australian Indigenous smoking policy study. Front Public Health. 2016;4:228. doi:10.3389/fpubh.2016.00228
293. Waddell C, Lavis JN, Abelson J, et al. Research use in children's mental health policy in Canada: maintaining vigilance amid ambiguity. Soc Sci Med. 2005;61(8):1649-1657. doi:10.1016/j.socscimed.2005.03.032

294. Walls H, Liverani M, Chheng K, Parkhurst J. The many meanings of evidence: a comparative analysis of the forms and roles of evidence within three health policy processes in Cambodia. Health Res Policy Syst. 2017;15(1):95. doi:10.1186/s12961-017-0260-2
295. Walugembe DR, Kiwanuka SN, Matovu JK, Rutebemberwa E, Reichenbach L. Utilization of research findings for health policy making and practice: evidence from three case studies in Bangladesh. Health Res Policy Syst. 2015;13:26. doi:10.1186/s12961-015-0015-x
296. Waqa G, Bell C, Snowdon W, Moodie M. Factors affecting evidence-use in food policy-making processes in health and agriculture in Fiji. BMC Public Health. 2017;17(1):51. doi:10.1186/s12889-016-3944-6
297. Waqa G, Moodie M, Snowdon W, et al. Exploring the dynamics of food-related policymaking processes and evidence use in Fiji using systems thinking. Health Res Policy Syst. 2017;15(1):74. doi:10.1186/s12961-017-0240-6
298. Weatherly H, Drummond M, Smith D. Using evidence in the development of local health policies. Some evidence from the United Kingdom. Int J Technol Assess Health Care. 2002;18(4):771-781. doi:10.1017/s0266462302000582
299. Wehrens R, Bekker M, Bal R. The construction of evidence-based local health policy through partnerships: research infrastructure, process, and context in the Rotterdam 'Healthy in the City' programme. J Public Health Policy. 2010;31(4):447-460. doi:10.1057/jphp.2010.33
300. Wehrens R, Bekker M, Bal R. Coordination of research, policy and practice: a case study of collaboration in the field of public health. Sci Public Policy. 2011;38(10):755-766. doi:10.1093/spp/38.10.755
301. Wei Y, Pong RW, Shi L, et al. Perceptions of health technology assessment knowledge translation in China: a qualitative study on HTA researchers and policy-makers. International Journal of Healthcare Technology and Management. 2017;16(1-2):44-58. doi:10.1504/ijhtm.2017.087599
302. Weiss CH, Murphy-Graham E, Birkeland S. An alternate route to policy influence: how evaluations affect DARE. Am J Eval. 2005;26(1):12-30. doi:10.1177/1098214004273337
303. Whiteford HA, Meurk C, Carstensen G, Hall W, Hill P, Head BW. How did youth mental health make it onto Australia's 2011 Federal Policy Agenda? Sage Open. 2016;6(4):2158244016680855. doi:10.1177/2158244016680855
304. Whiteside A, Henry FE. The impact of HIV and AIDS research: a case study from Swaziland. Health Res Policy Syst. 2011;9(Suppl 1):S9. doi:10.1186/1478-4505-9-s1-s9
305. Wild K, Kelly P, Barclay L, Martins N. Agenda setting and evidence in maternal health: connecting research and policy in Timor-Leste. Front Public Health. 2015;3:212. doi:10.3389/fpubh.2015.00212
306. Wilkinson K. Organised chaos: an interpretive approach to evidence-based policy making in Defra. Polit Stud. 2011;59(4):959-977. doi:10.1111/j.1467-9248.2010.00866.x
307. Williams I, McIver S, Moore D, Bryan S. The use of economic evaluations in NHS decision-making: a review and empirical investigation. Health Technol Assess. 2008;12(7):iii, ix-x, 1-175. doi:10.3310/hta12070
308. Willmott M, Womack J, Hollingworth W, Campbell R. Making the case for investment in public health: experiences of Directors of Public Health in English local government. J Public Health (Oxf). 2016;38(2):237-242. doi:10.1093/pubmed/fdv035

309. Witter S, Kardan A, Scott M, Moore L, Shaxson L. Generating demand for and use of evaluation evidence in government health ministries: lessons from a pilot programme in Uganda and Zambia. Health Res Policy Syst. 2017;15(1):86. doi:10.1186/s12961-017-0250-4
310. Woelk G, Daniels K, Cliff J, et al. Translating research into policy: lessons learned from eclampsia treatment and malaria control in three southern African countries. Health Res Policy Syst. 2009;7:31. doi:10.1186/1478-4505-7-31
311. Wu S, Legido-Quigley H, Spencer J, Coker RJ, Khan MS. Designing evaluation studies to optimally inform policy: what factors do policy-makers in China consider when making resource allocation decisions on healthcare worker training programmes? Health Res Policy Syst. 2018;16(1):16. doi:10.1186/s12961-018-0292-2
312. Wye L, Brangan E, Cameron A, Gabbay J, Klein JH, Pope C. Evidence based policy making and the 'art' of commissioning - how English healthcare commissioners access and use information and academic research in 'real life' decision-making: an empirical qualitative study. BMC Health Serv Res. 2015;15:430. doi:10.1186/s12913-015-1091-x
313. Wyndham-West M, Wiktorowicz M, Tsasis P. Power and culture in emerging medical technology policymaking: the case of the human papillomavirus (HPV) vaccine in Canada. Evid Policy. 2018;14(2):277-299. doi:10.1332/174426417x14845753387144
314. Yanovitzky I, Weber M. Analysing use of evidence in public policymaking processes: a theory-grounded content analysis methodology. Evid Policy. 2020;16(1):65-82. doi:10.1332/174426418x15378680726175
315. Yazdizadeh B, Mohtasham F, Velayati A. Impact assessment of Iran's health technology assessment programme. Health Res Policy Syst. 2018;16(1):15. doi:10.1186/s12961-018-0286-0
316. Young I, Gropp K, Pintar K, et al. Experiences and attitudes towards evidence-informed policy-making among research and policy stakeholders in the Canadian agri-food public health sector. Zoonoses Public Health. 2014;61(8):581-589. doi:10.1111/zph.12108
317. Young T, Shearer JC, Naude C, Kredo T, Wiysonge CS, Garner P. Researcher and policymaker dialogue: the Policy BUDDIES Project in Western Cape Province, South Africa. BMJ Glob Health. 2018;3(6):e001130. doi:10.1136/bmjgh-2018-001130
318. Yousefinezhadi T, Mosadeghrad AM, Arab M, Ramezani M, Sari AA. An analysis of hospital accreditation policy in Iran. Iran J Public Health. 2017;46(10):1347-1358.
319. Zechmeister I, Schumacher I. The impact of health technology assessment reports on decision making in Austria. Int J Technol Assess Health Care. 2012;28(1):77-84. doi:10.1017/s0266462311000729
